# Supplementary material for: Benchmarking the negatives: Effect of negative data generation on the classification of miRNA-mRNA interactions
Source: PLoS Comput Biol. 2024 Aug 26;20(8):e1012385. doi: 10.1371/journal.pcbi.1012385 (PMC11379385; doi:10.1371/journal.pcbi.1012385)
Supplement: S1 Text — (PDF) [file pcbi.1012385.s001.pdf]

Benchmarking the negatives: effect of negative data generation on the classification of miRNA-mRNA interactions

Efrat Cohen-Davidi and Isana Veksler-Lublinsky

1 Supplementary figures

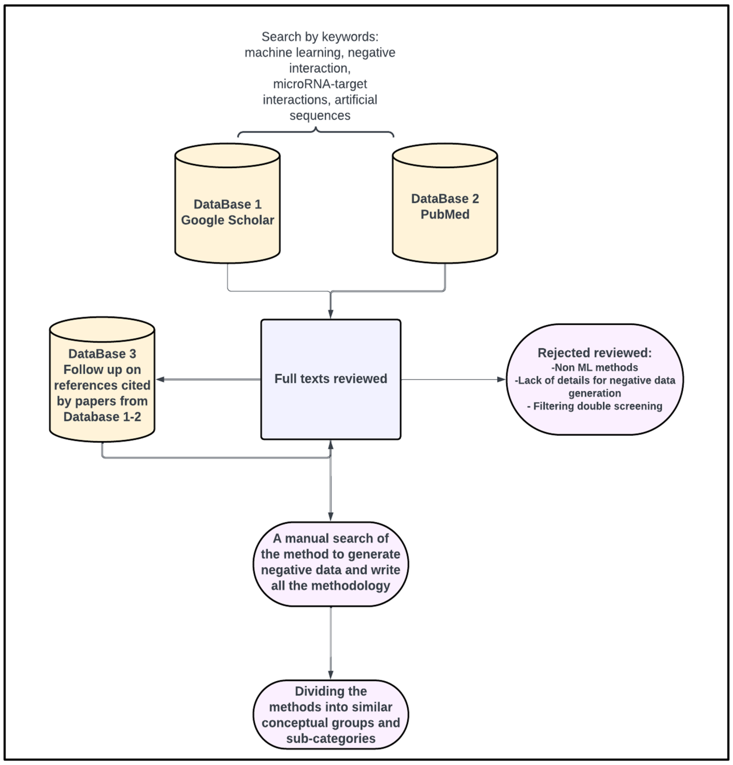

Fig A. Literature search flow diagram. To initiate our study, we formulated a precise research question and identified a set of relevant keywords. Using two main search engines, Google Scholar and PubMed, we conducted searches for papers related to our research question. Subsequently, we examined each paper to assess its relevance. If any of the papers cited additional relevant works, we also reviewed them (Database 3). After compiling a list of pertinent papers, we systematically extracted the methods employed for generating negative interactions. We categorized these methods into groups, each representing a collection of similar techniques with minor distinctions among the sub-methods.

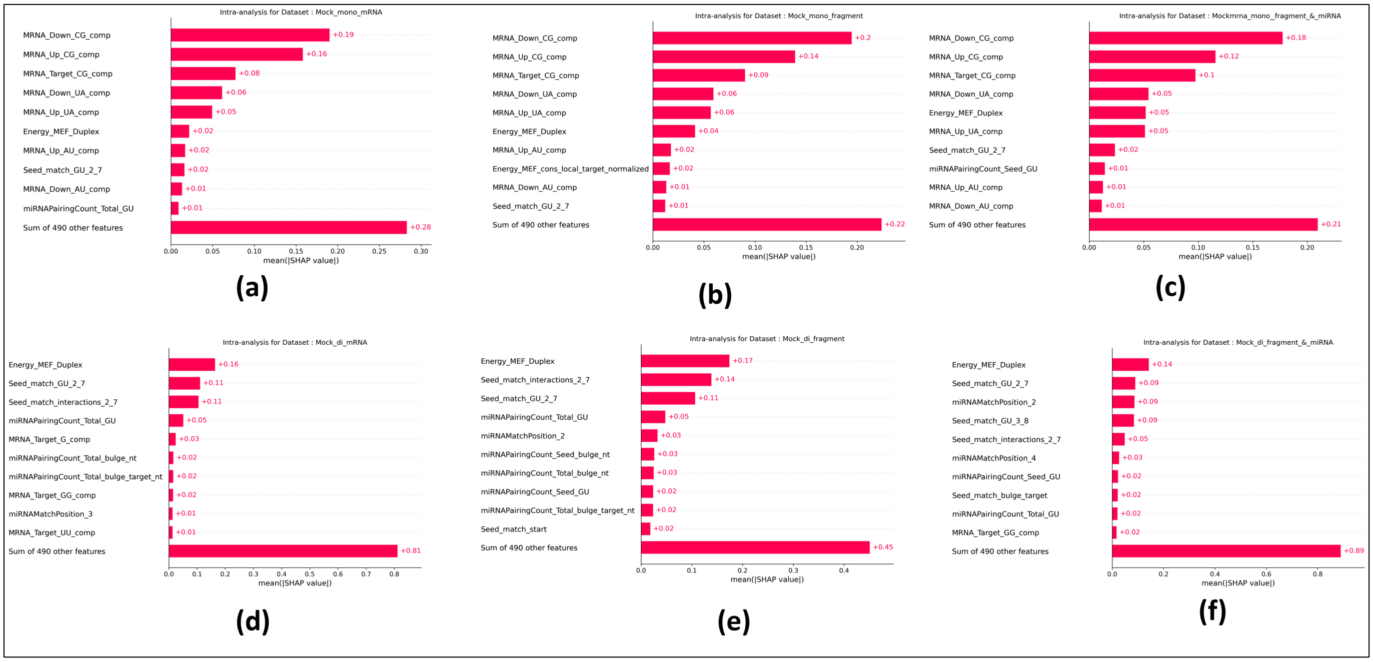

Fig B. SHAP importance for different *Mock mRNA* datasets in intra-dataset analysis. Feature importance for models trained and tested on *Mock mRNA* datasets: (a)*Mock_mono_mRNA*, (b) *Mock_mono_fragment*, (c) *Mock_mono_fragment_&_miRNA*, (d) *Mock_di_mRNA*, (e) *Mock_di_fragment* and (f) *Mock_di_fragment_&_miRNA* are shown. The top ten features are ordered by their importance for the model's predictions. Features ending with "comp" are associated with the mRNA composition within the target site or its flanking sequences.

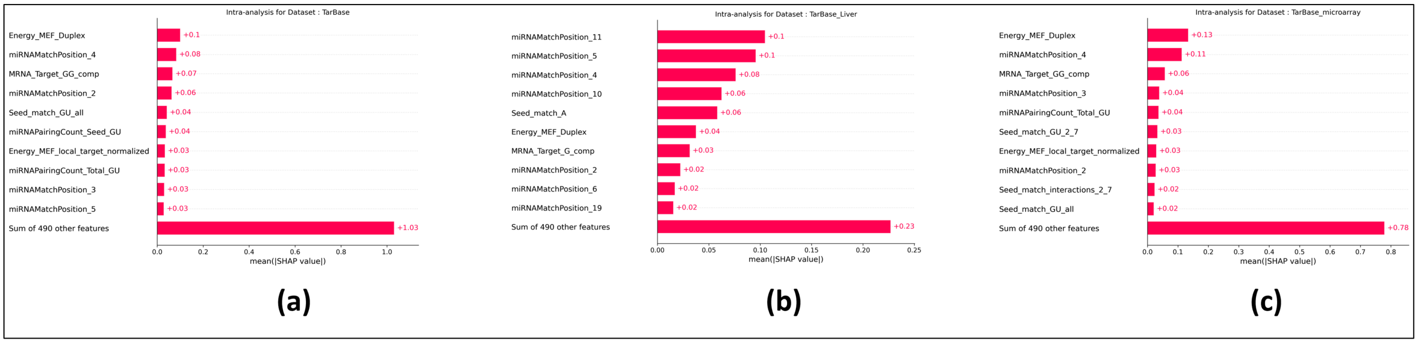

Fig C. SHAP importance for different *TarBase* datasets. Feature importance for models trained and tested on (a) *TarBase*, (b) *TarBase_Liver*, and (c) *TarBase_microarray* datasets. The top ten features are ordered by their importance for the model's predictions.

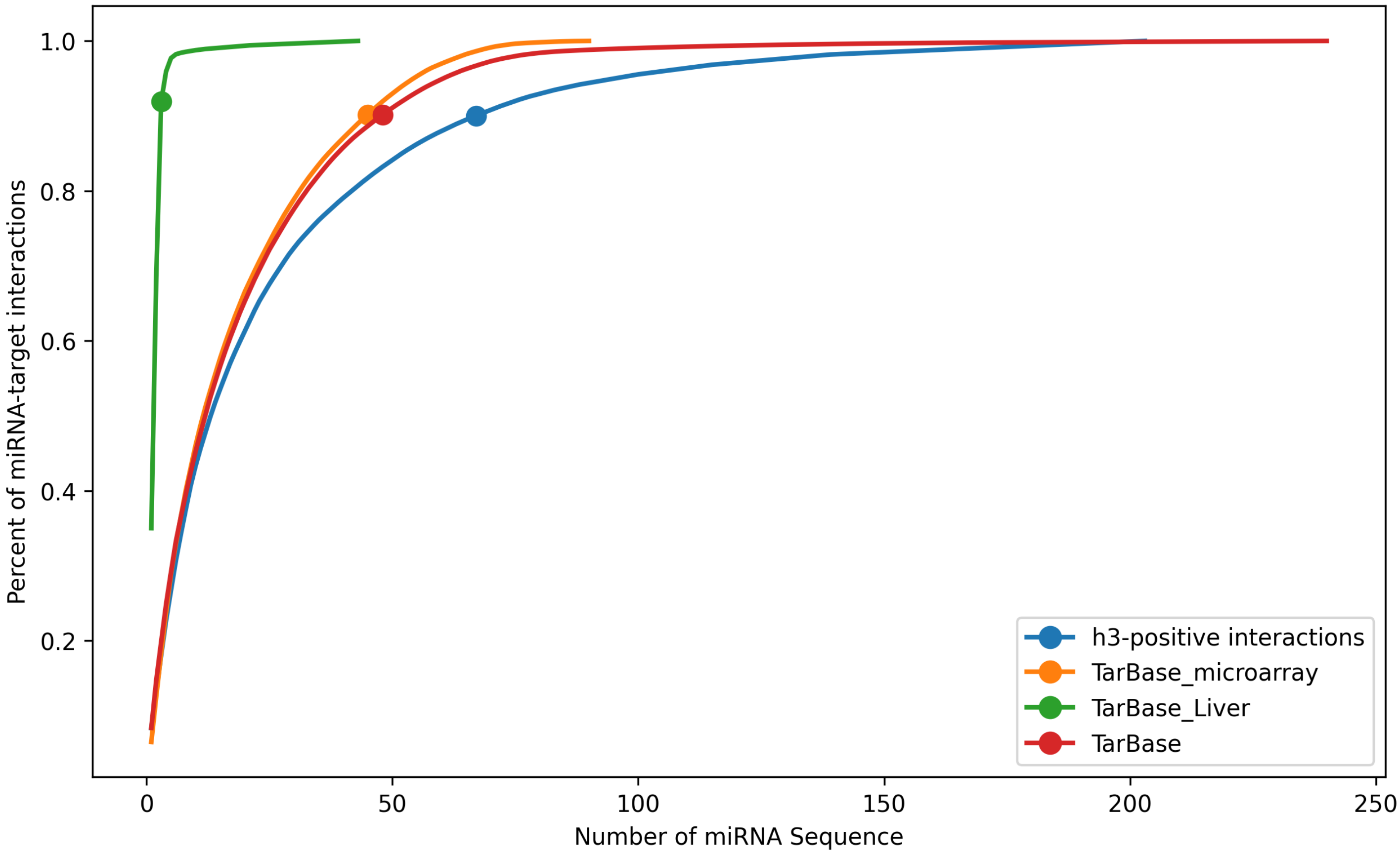

Fig D. Cumulative sum of miRNA appearances in different TarBase datasets. Each curve corresponds to the cumulative sum of one of the datasets (*TarBase*, *TarBase_Liver*, *TarBase_microarray* and *h3*), where the minimum number of unique miRNA sequences needed to represent 90% of the interactions within the dataset is indicated by a filled circle. The curves were normalized to reach the same height. The width of each curve represents the number of unique miRNA sequences that comprise it.

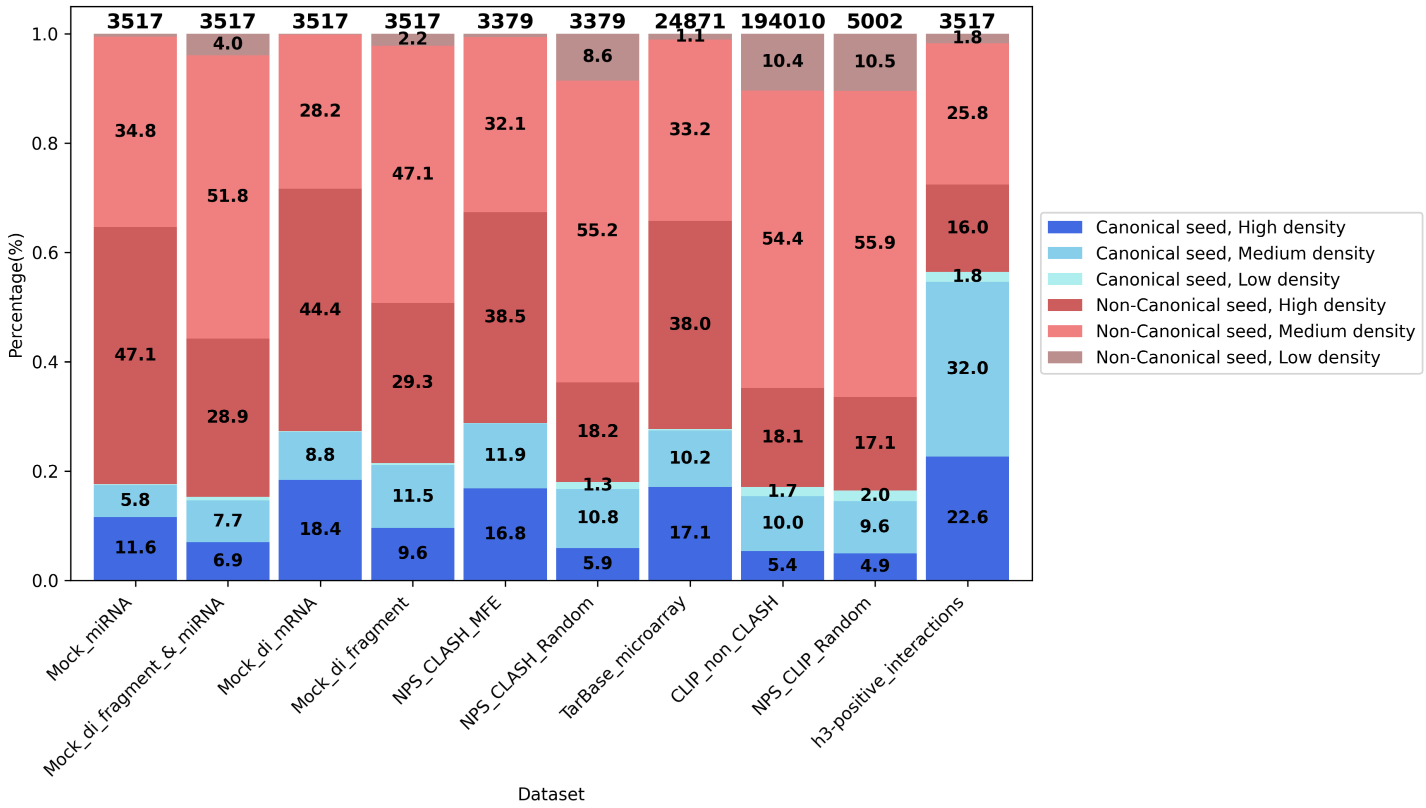

Fig E. Classifications of the miRNA-target duplexes, based on their base-pairing patterns. Distribution of miRNA-target duplexes according to the seed type (canonical or non-canonical) and the base-pairing density (low: <11 bp, medium:11-16 bp, or high: >16bp). The number above each bar indicates the total number of interactions in the dataset.

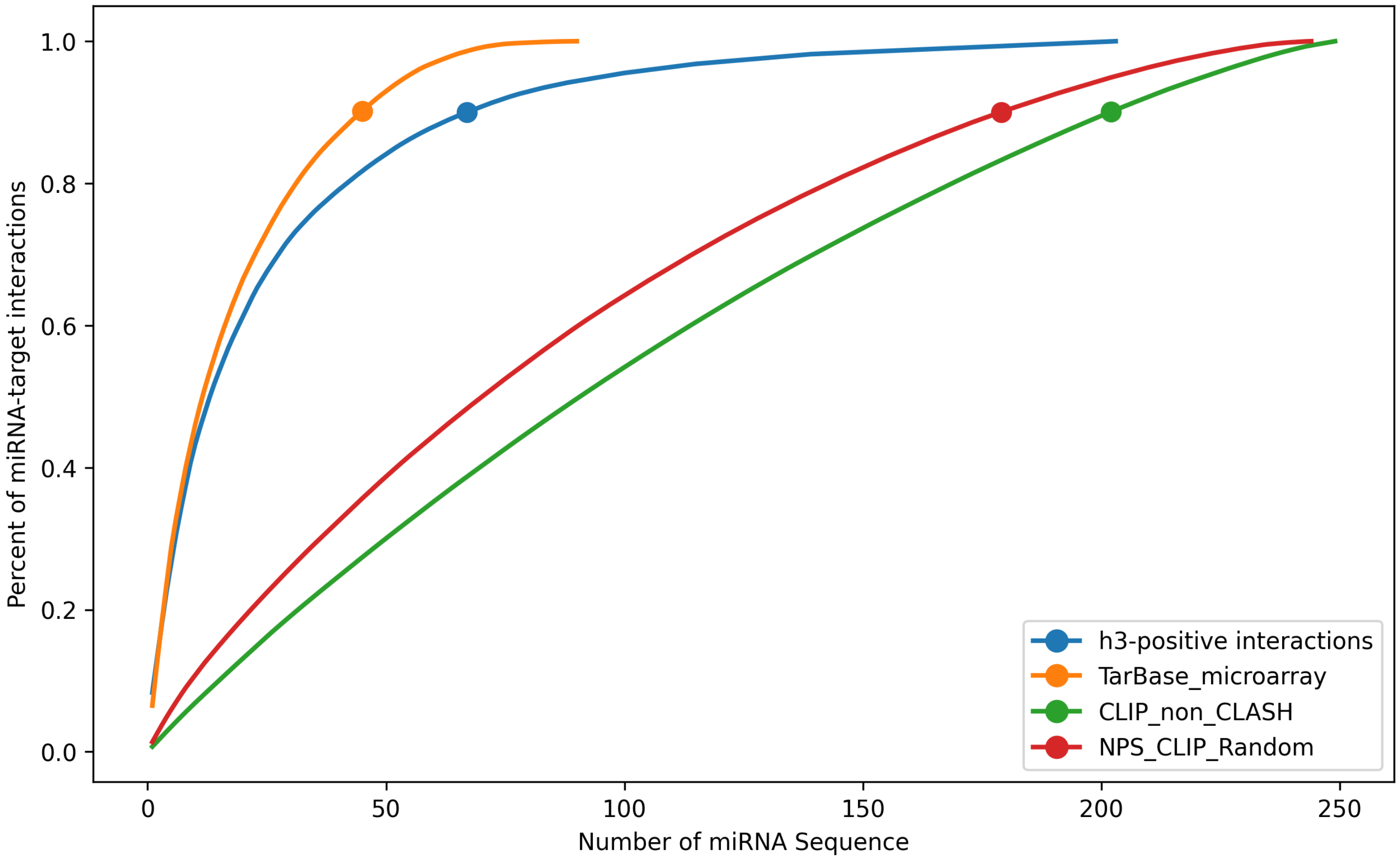

Fig F. Cumulative sum of miRNA appearances in negative datasets. Each curve corresponds to the cumulative sum of one of the datasets (*TarBase_microarray*, *CLIP_non_CLASH*, *NPS_CLIP_Random* and *h3*), where the minimum number of unique miRNA sequences needed to represent 90% of the interactions within the dataset is indicated by a filled circle. The curves were normalized to reach the same height. The width of each curve represents the number of unique miRNA sequences that comprise it. miRNAs found in *h3* correspond to miRNAs in datasets *Mock_di_fragment*, *Mock_di_mRNA*,*NPS_CLASH_MFE*, and *NPS_CLASH_Random* datasets.

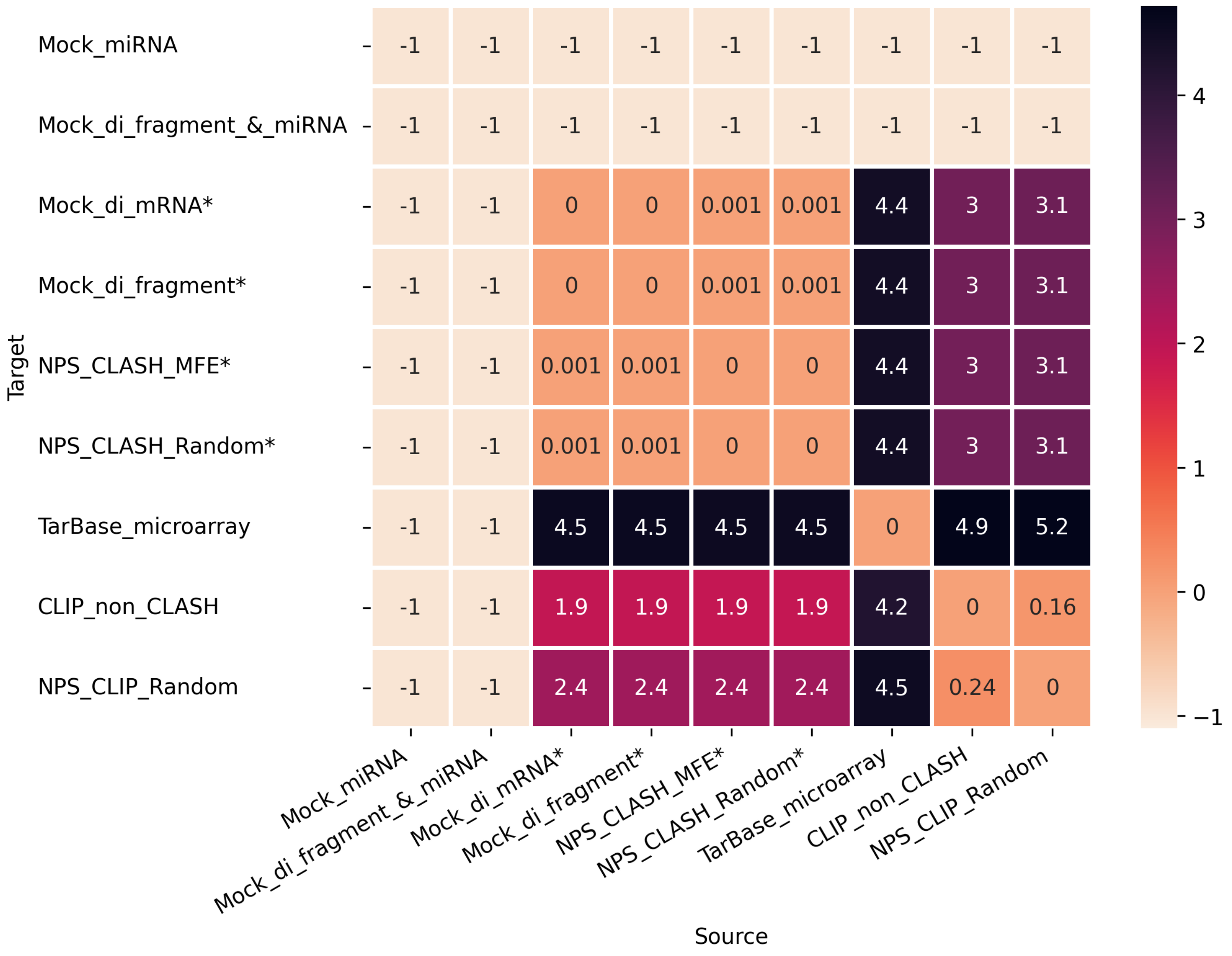

Fig G. Kullback–Leibler (KL) divergence of all dataset pairs. Each cell *(j,i)* represents the divergence from a source dataset i to a target dataset j (KL(j || i)), based on their miRNA sequence distributions. Datasets marked with asterisks have the same miRNA distribution. Datasets that are based on artificial miRNAs were not evaluated and are marked with -1.

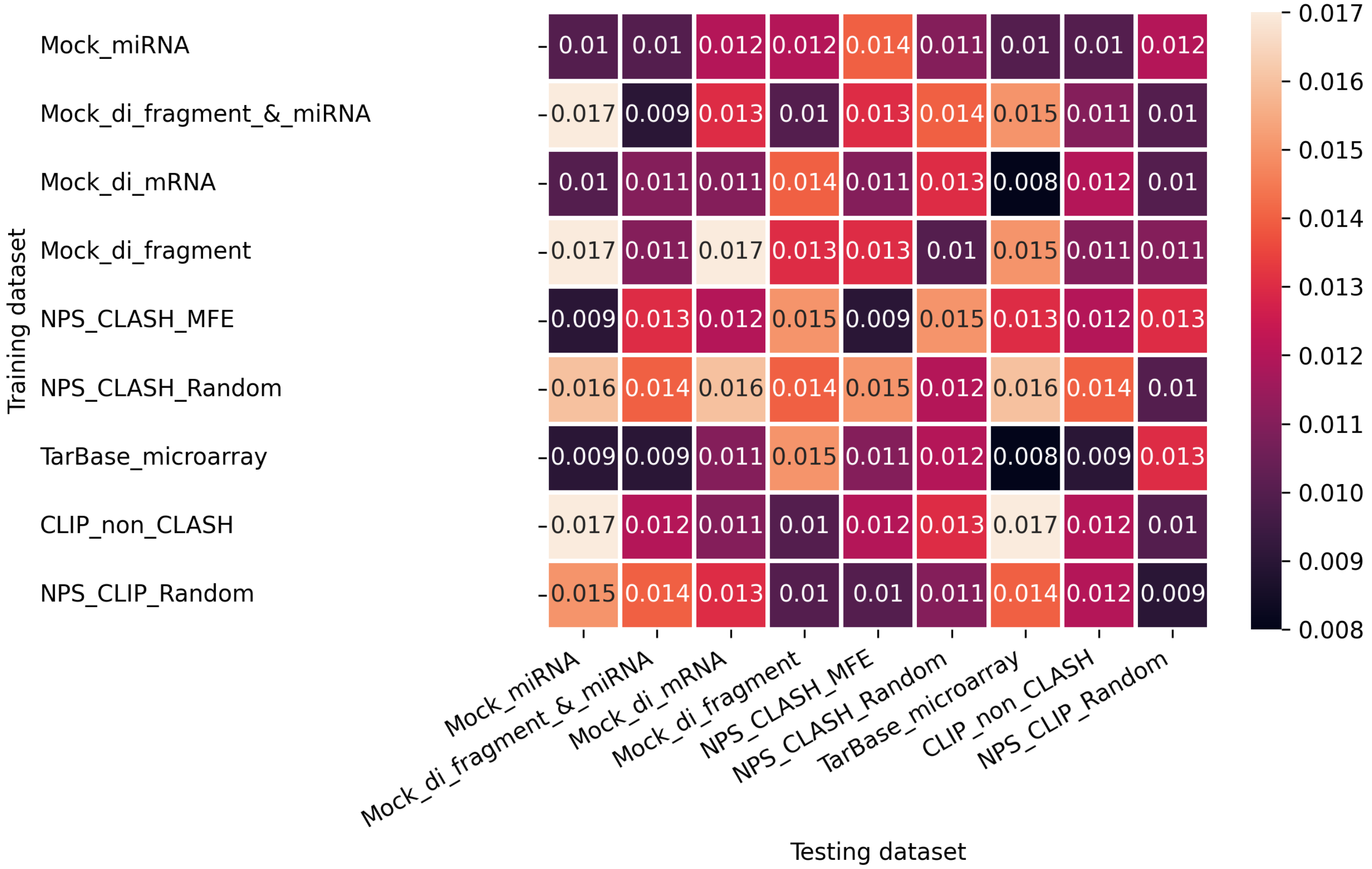

Fig H. The standard deviation (std) values of cross-datasets classification accuracy (ACC). Each cell *(i,j)* represents the std of ACC of the 20 classifiers that were trained on dataset i and tested on dataset j.

.

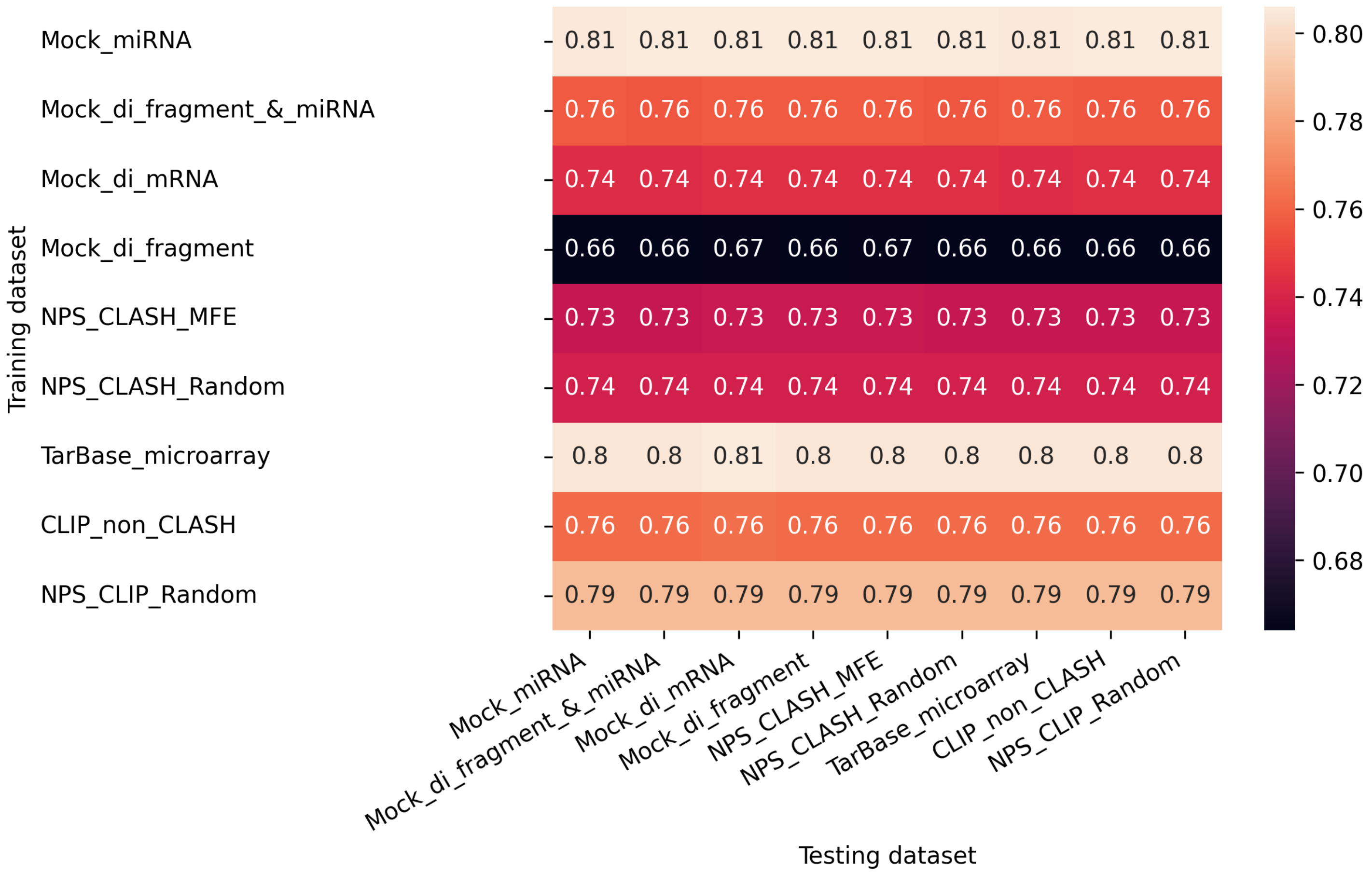

Fig I. The TPR of the evaluated models based on cross-learning datasets. Cross-dataset classification TPR is calculated for different negative dataset pairs. Each cell *(i,j)* represents the mean TPR of the 20 classifiers that were trained on 20 training-testing dataset splits of dataset i and tested on dataset j.

.

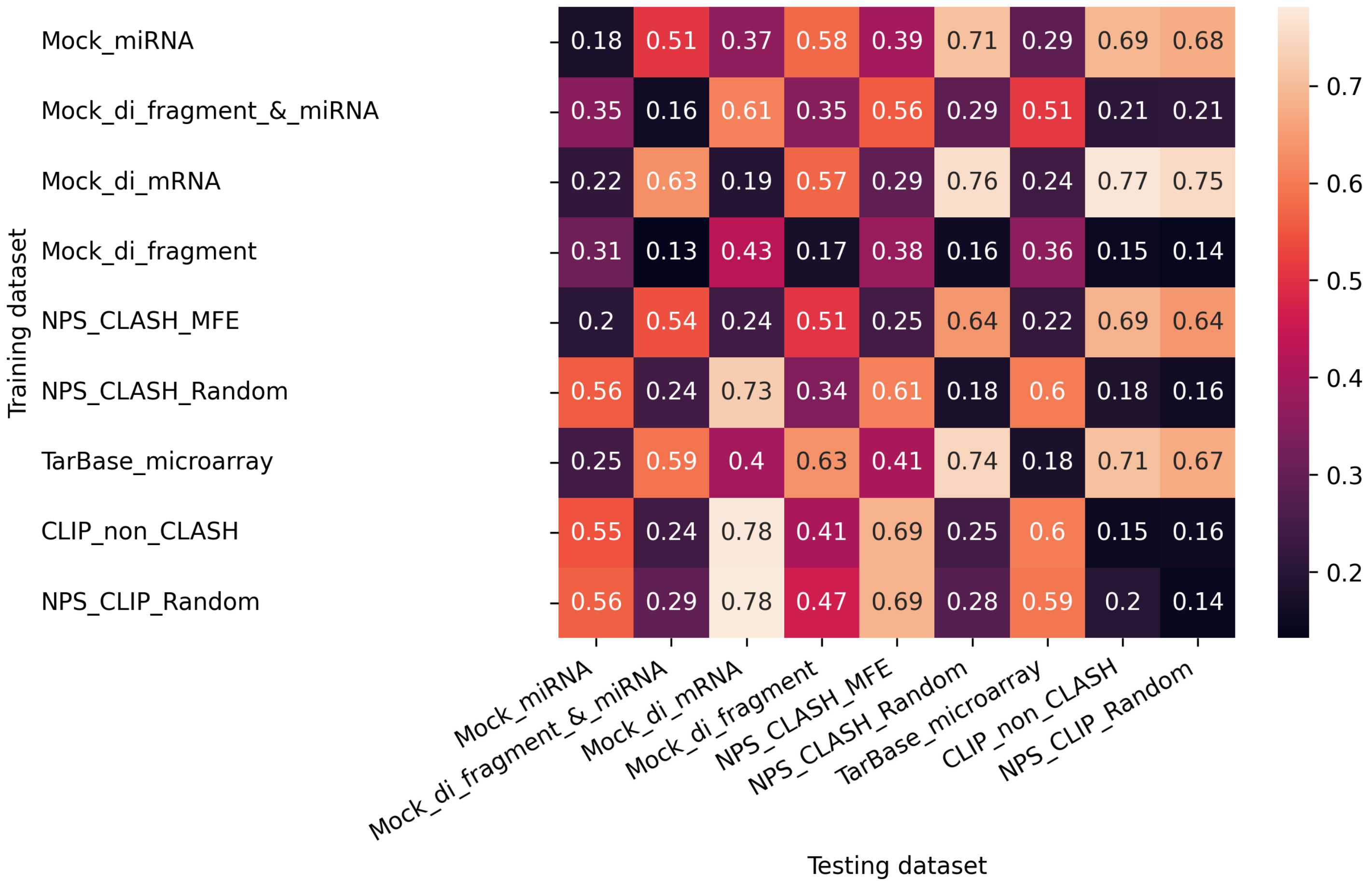

Fig J. The FPR of the evaluated models based on cross-learning datasets. Cross-dataset classification FPR is performed with different negative dataset pairs. Each cell *(i,j)* represents the mean FPR of the 20 classifiers that were trained on 20 training-testing dataset splits of dataset i and tested on dataset j.

.

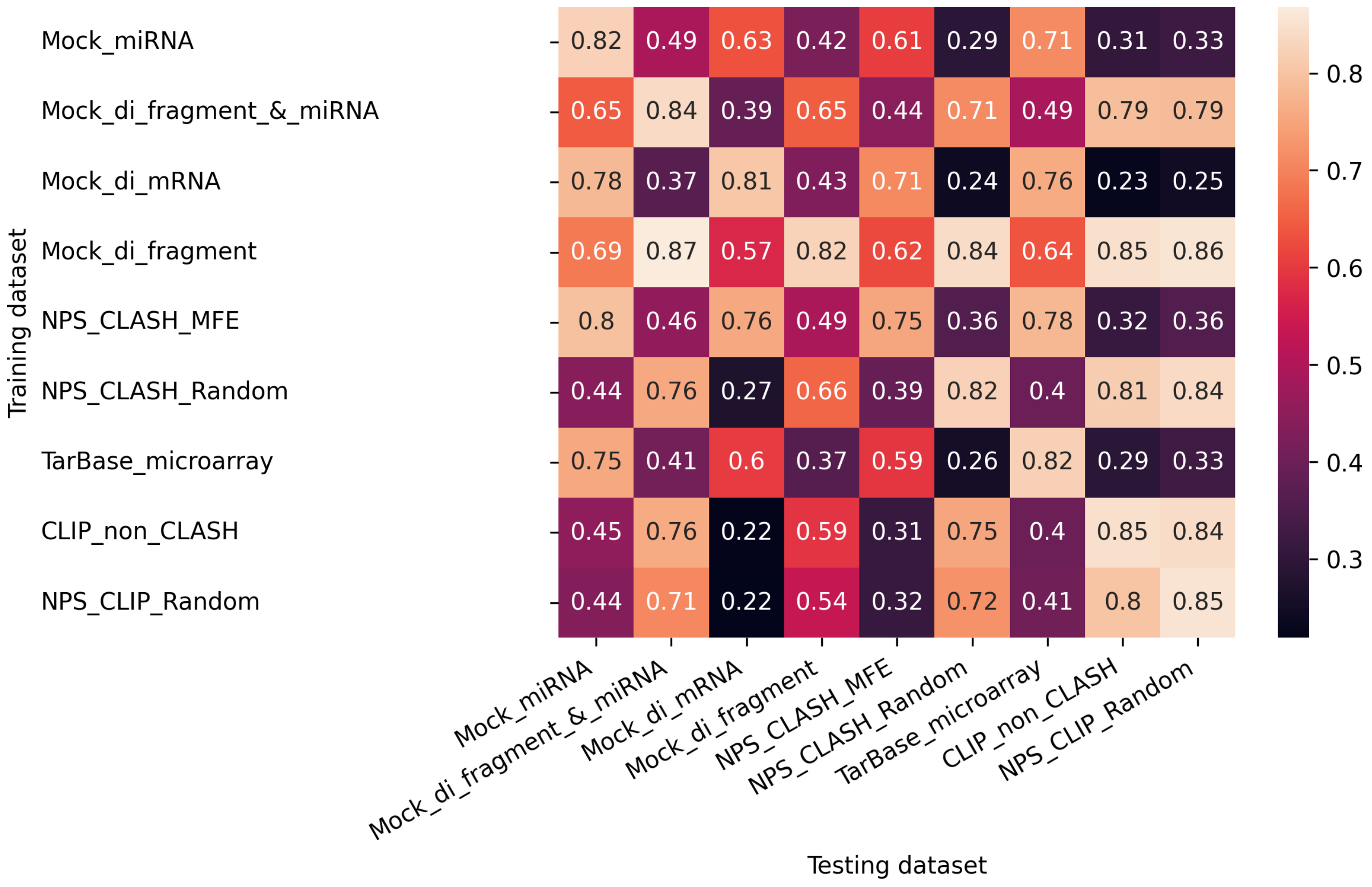

	Mock_miRNA	Mock_di_fragment_&_miRNA	Mock_di_mRNA	Mock_di_fragment	NPS_CLASH_MFE	NPS_CLASH_Random	TarBase_microarray	CLIP_non_CLASH	NPS_CLIP_Random
Mock_miRNA	0.82	0.49	0.63	0.42	0.61	0.29	0.71	0.31	0.33
Mock_di_fragment_&_miRNA	0.65	0.84	0.39	0.65	0.44	0.71	0.49	0.79	0.79
Mock_di_mRNA	0.78	0.37	0.81	0.43	0.71	0.24	0.76	0.23	0.25
Mock_di_fragment	0.69	0.87	0.57	0.82	0.62	0.84	0.64	0.85	0.86
NPS_CLASH_MFE	0.8	0.46	0.76	0.49	0.75	0.36	0.78	0.32	0.36
NPS_CLASH_Random	0.44	0.76	0.27	0.66	0.39	0.82	0.4	0.81	0.84
TarBase_microarray	0.75	0.41	0.6	0.37	0.59	0.26	0.82	0.29	0.33
CLIP_non_CLASH	0.45	0.76	0.22	0.59	0.31	0.75	0.4	0.85	0.84
NPS_CLIP_Random	0.44	0.71	0.22	0.54	0.32	0.72	0.41	0.8	0.85

Fig K. The TNR of the evaluated models based on cross-learning datasets. Cross-dataset classification TNR is performed with different negative dataset pairs. Each cell *(i,j)* represents the mean TNR of the 20 classifiers that were trained on 20 training-testing dataset splits of dataset i and tested on dataset j.

.

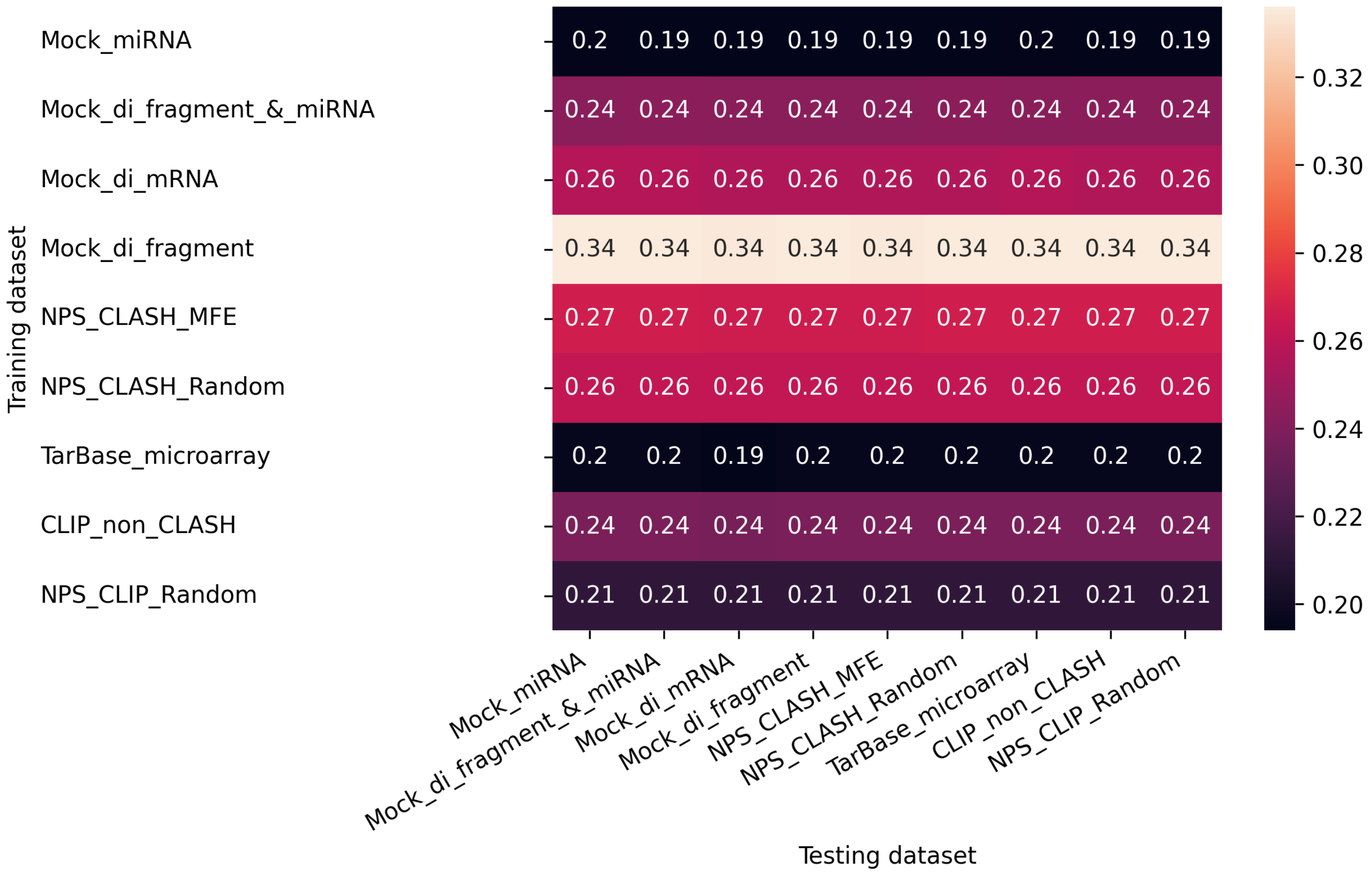

Fig L. The FNR of the evaluated models based on cross-learning datasets. Cross-dataset classification FNR is performed with different negative dataset pairs. Each cell *(i,j)* represents the mean FNR of the 20 classifiers that were trained on 20 training-testing dataset splits of dataset i and tested on dataset j.

.

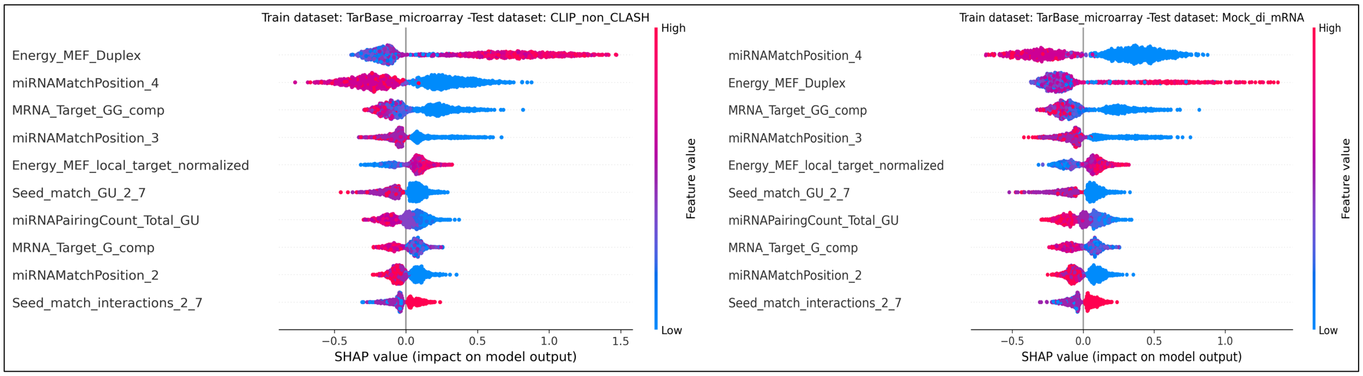

Fig M. SHAP summary plots for cross-datasets classification. Summary plot for the pairs (*TarBase_microarray*, *CLIP_non_CLASH*) and (*TarBase_microarray*, *Mock_di_mRNA*) showing the top ten features in order of importance.

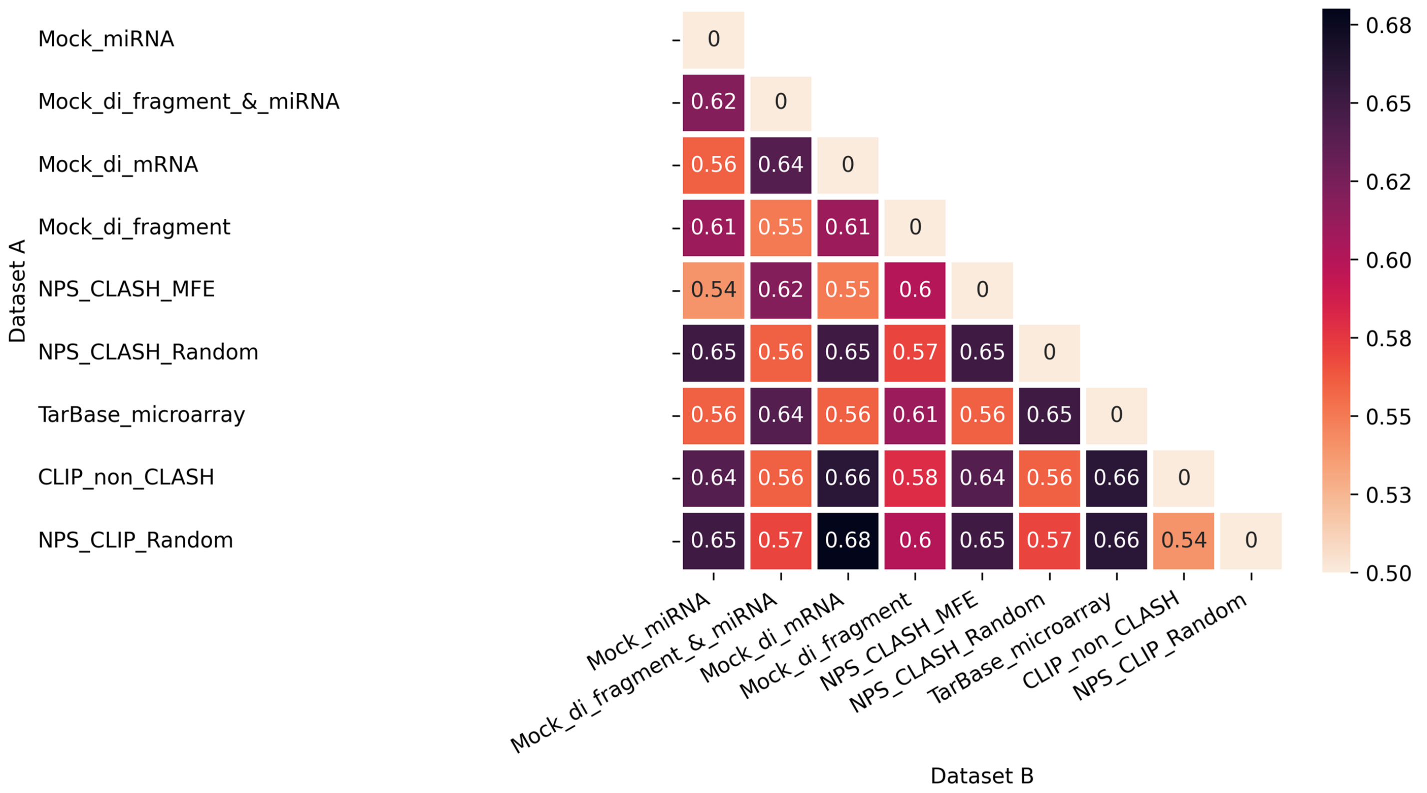

Fig N. Two negative-classes classification results. Two negative-classes classification ACC metric for pairs of negative datasets. Each cell *(A,B)* represents the ACC of a classifier that was trained and tested on negative interactions from datasets A and B (labeled as "1" and "0" respectively). The classification ACC varies among the pairs, ranging from 0.54 to 0.68.

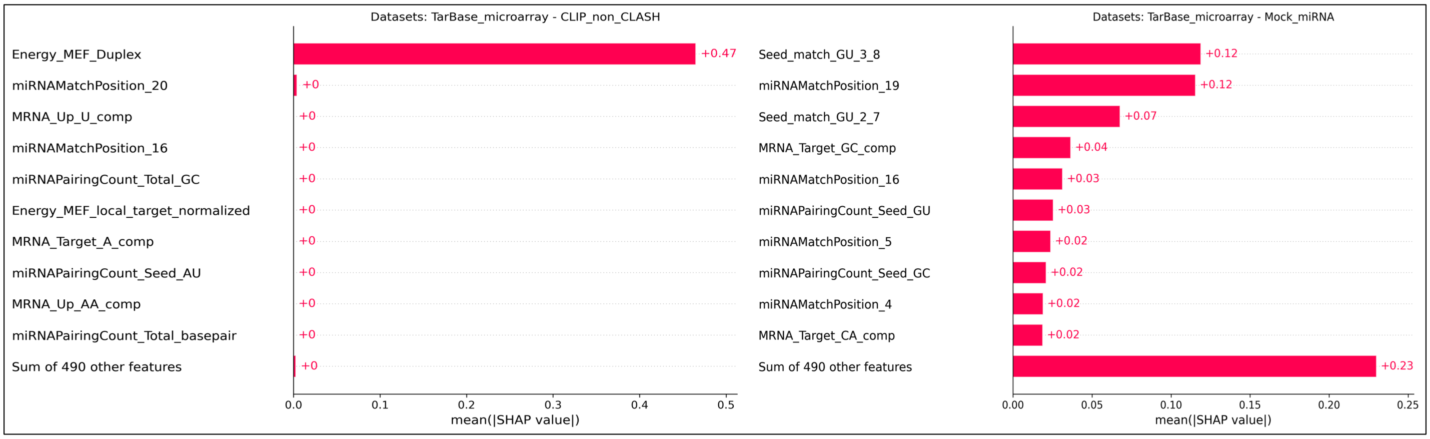

Fig O. SHAP importance for two negative-classes pairs. The feature importance summary for (*Tarbase_microarray*, *CLIP_non_CLASH*) and (*Tarbase_microarray*, *Mock_miRNA*) showing the top ten features in their order of importance. The plot for the pair on the right exhibited less distinct separation while the plot for the pair on the left shows that the energy of the duplex was the dominant feature that significantly impacted the model.

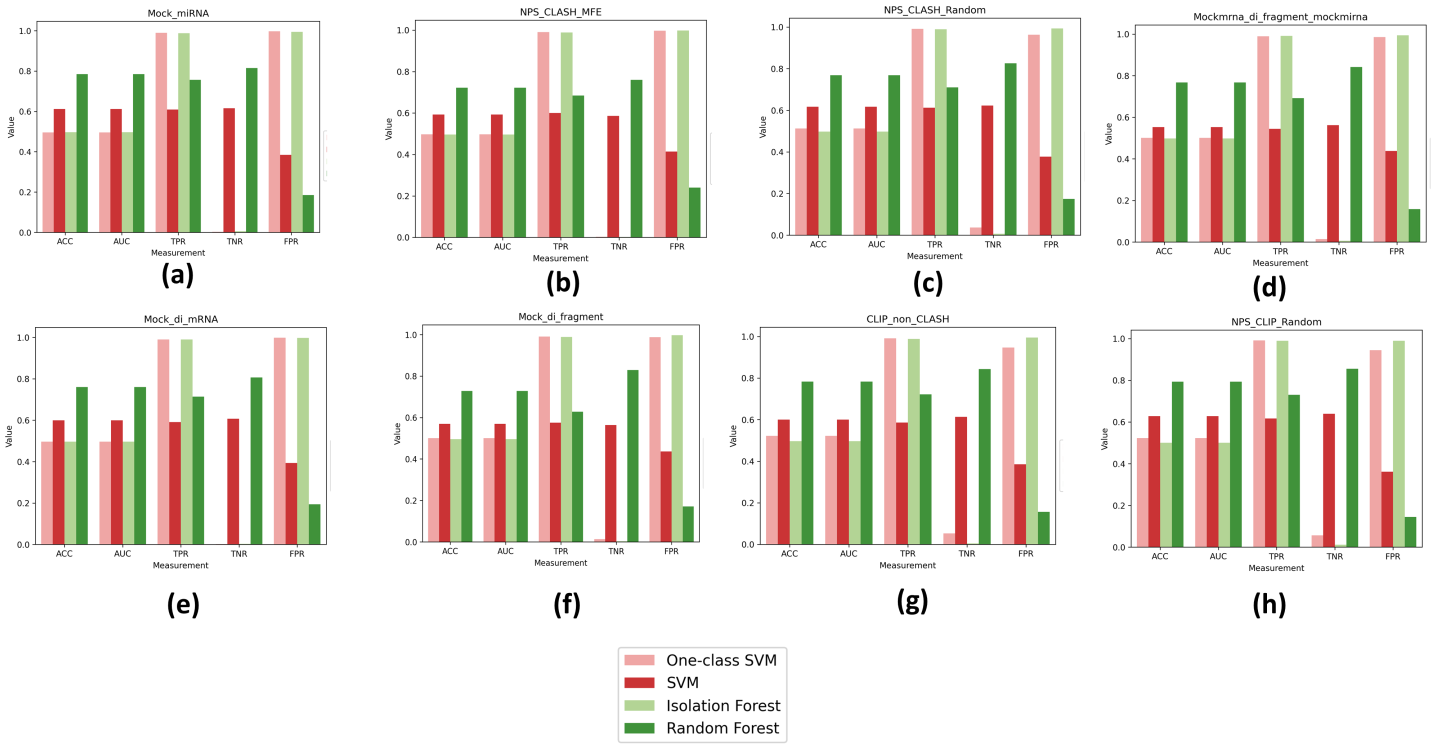

Fig P. Comparing the performance of One-class classifiers with their respective binary classifier. The results are organized into panels (a-h), such that each panel shows a different negative dataset. The performance of four classifiers is measured by ACC, AUC, TPR, TNR, and FPR. The One-class SVM and Isolation Forest were trained on positive data only, while the SVM and Random Forest were trained on both positive and negative datasets. All four models were tested for their ability to classify positive and negative interactions. Each panel (a-h) reported on a different negative dataset.

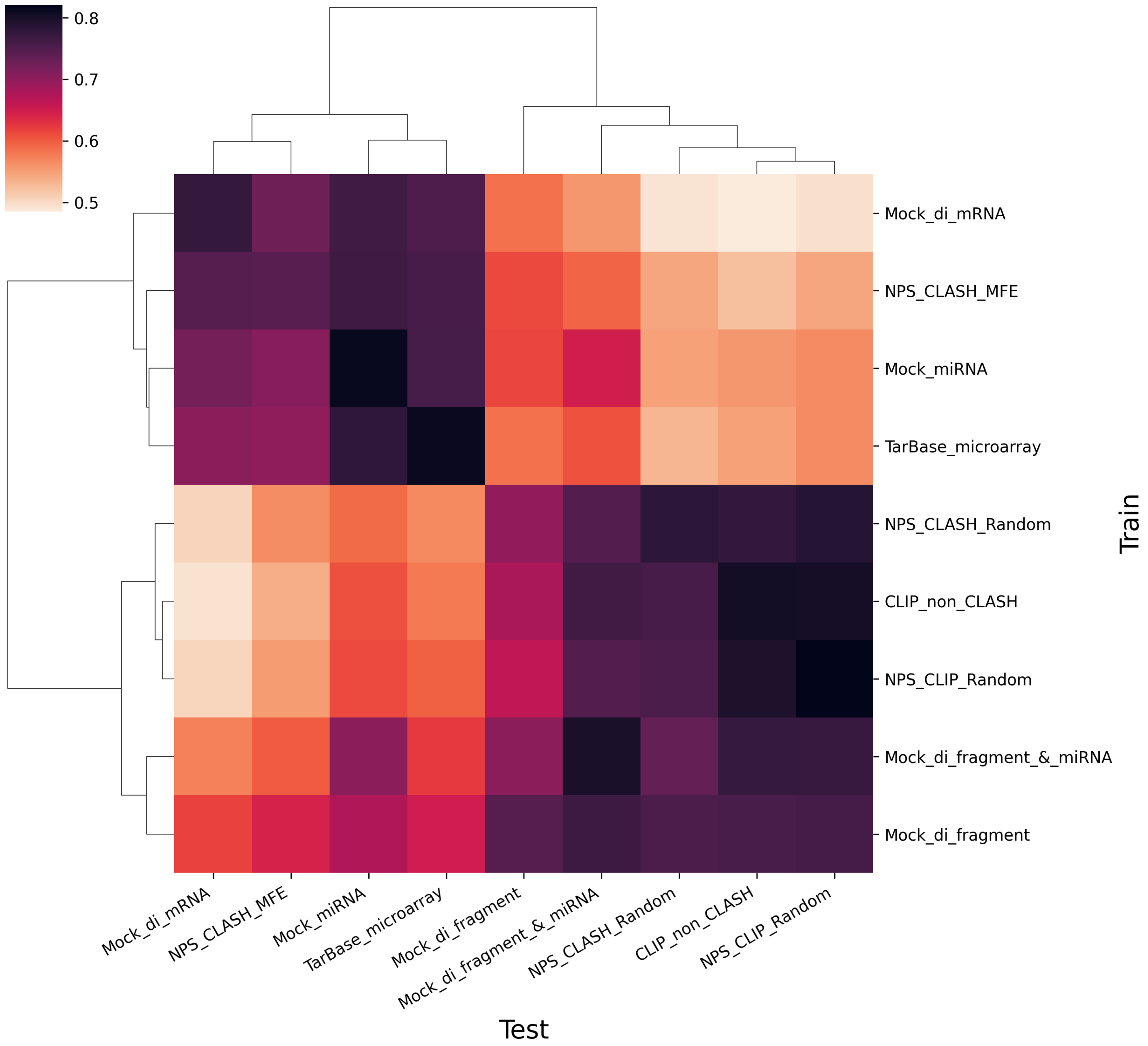

Fig Q. Clustering of datasets based on their performance in the cross-datasets classification. The clustering of the datasets based on the ACC values clearly indicates the presence of two distinct groups. Within each group, the performance of the datasets is similar.

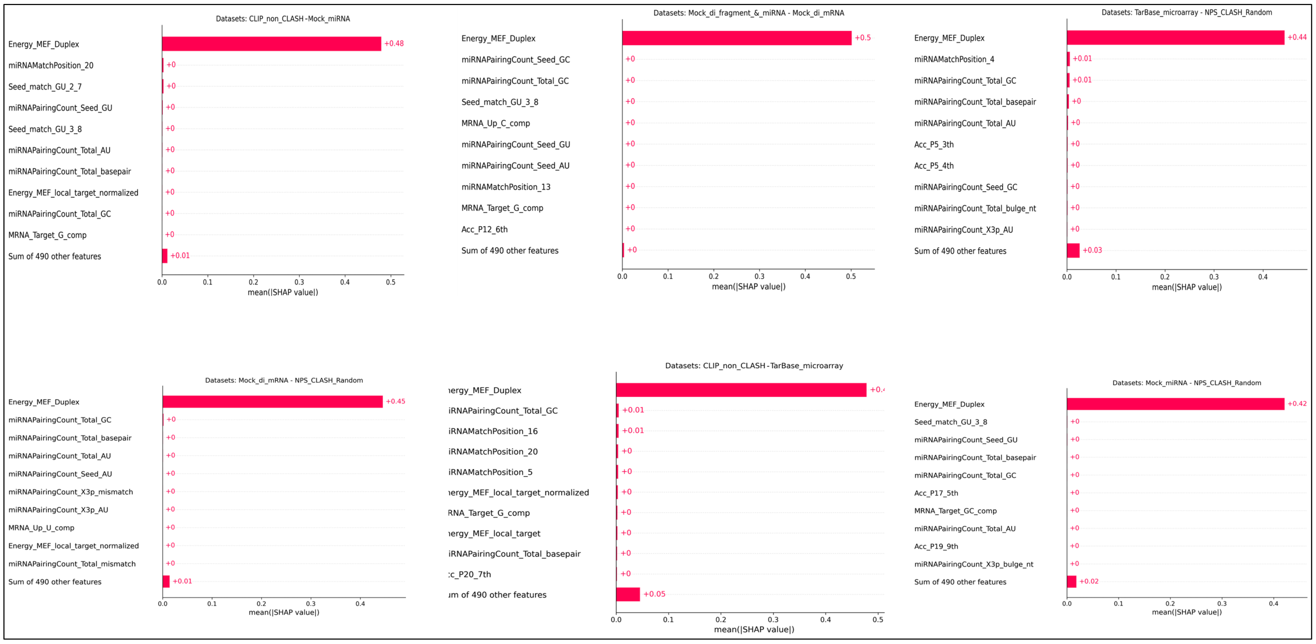

Fig R. SHAP importance for two negative-classes pairs. The feature importance summary for classifiers tested for their ability to distinguish between pairs of negative datasets, showing the top ten features in their order of importance. All plots show that the energy characteristic emerges as the most influential factor in the classification process.

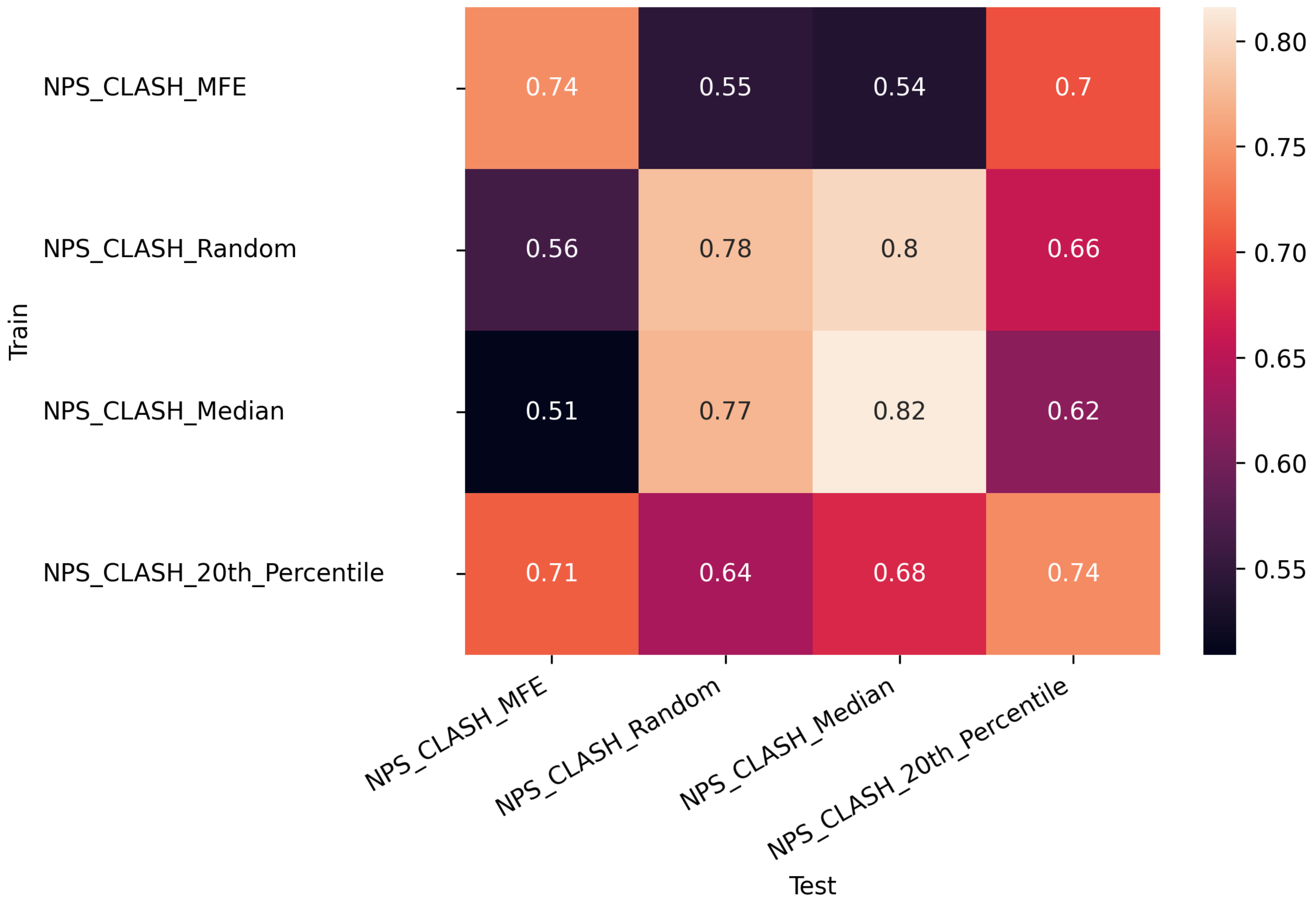

Fig S. Cross-dataset classification results for *NPS_CLASH* datasets. Cross-dataset classification ACC metric for NPS_CLASH negative datasets with different filtering options. For each miRNA-target pair, all candidates were ranked by the energy of the miRNA-site duplex, and the candidate with the lowest MFE, random MFE, median MFE, or random selection from the MFEs in the 20th percentile was chosen for the negative dataset (*NPS_CLASH_MFE*, *NPS_CLASH_Random*, *NPS_ClASH_Median* and *NPS_ClASH_20th_Percentile*). Each cell *(i,j)* represents the mean ACC of the 20 classifiers that were trained on dataset i and tested on dataset j.

.

2 Supplementary Text

2.1 Features

To represent miRNA-target interactions, we used 500 expert-designed features, classified into two categories (high level and low level) and five subcategories (Table 4 in the main text). 490 features were adopted from[3] and the remaining 10 features that characterize miRNA pairing were added in this study. For a full description of the features, see Table S1.

The *seed features* describe the base-pairing characteristics of the seed region (positions 1–8 on the miRNA). This representation includes 13 features: three features describe the number of interactions in [nt1–8, nt2–7, and nt3–8]; three features describe the number of GUs in [nt1–8, nt2–7, and nt3–8]; three features provide information about the number of mismatches (before the first match, inside the seed, and after the last match in the seed region); two features describe the number of bulges (miRNA side and target side); and two features address additional properties (starts with A and index of the first base-pair).

The *free energy* category includes seven features representing the minimum free energy of the miRNA–mRNA duplex and the mRNA sequence at different regions, including seed, non-seed, site, and flanking regions.

The *mRNA composition* category consists of 62 features that provide information regarding the target mRNA, namely, the distance of the site from the edges of the 3'UTR (two features), 1- and 2-mer sequence composition within the site region (20 features), and 1- and 2-mer sequence composition of the up and down 70nt flanking region (20 features each).

The *miRNA pairing* category consists of 48 features that describe the duplex itself, including information about base-pairs in each location of the miRNA (20 features) and a total count of base-pairs, mismatches and gaps in the site region (28

features).

The *site accessibility* features were calculated for each 3'UTR sequence containing the seed site, using RNAplfold in the ViennaRNA package[1] with the following parameters: *winsize = 80*, *span = 40*, and *ulength = 10*, as was suggested by previous works.[2,4] The output of RNAplfold provided, for each nucleotide, the mean probability that regions of lengths 1–10 (ulength), ending at this nucleotide are unpaired. Of these calculations, we considered only the region that corresponds to the seed region on the target mRNA (p2–p8) with 15 flanking bases to either side (37 bases in total), resulting in $37 \times 10 = 370$ features.

3 Pseudocode for the negative data generation methods

3.1 Mock miRNA

Algorithm 1 Mock miRNA Dataset

```
Input: Positive miRNA–mRNA interactions dataset
Output: Mock_miRNA negative interactions dataset
for each record ∈ positive data do
    repeat
        repeat
            shuffled_miRNA←uShuffle (record.miRNAseq, k=1)
        until the sequence at positions 2–7 and 3–8 does not match with any real human miRNA
            or the attempt limit is reached
        mRNA←record.mRNAseq
        duplex ← RNAduplex (mRNA, shuffled_miRNA)
        if duplex.valid then
            Add (mRNA, shuffled_miRNA) interaction to Mock_miRNA dataset
        end if
    until valid duplex is obtained or attempt limit is reached
end for

return Mock_miRNA
```

3.2 Mock mRNA

Algorithm 2a Mock mRNA Dataset

```
Input: Positive miRNA–mRNA interactions dataset
Input: Type of mRNA shuffling k_parameter (k=1 or k=2)
Output: Mock mRNA negative interactions dataset
for each record ∈ positive data do
    repeat
        shuffled_mRNA ← uShuffle (record.mRNAseq, k=k_parameter)
        miRNA←record.miRNAseq
        duplex ← RNAduplex (shuffled_mRNA, miRNA)
        if duplex.valid then
            Add (shuffled_mRNA, miRNA) interaction to Mock_mono_mRNA dataset if k = 1, or
                                                      Mock_di_mRNA dataset if k = 2
        end if
    until valid duplex is obtained or attempt is limit reached
end for

if k=1 then
    return Mock_mono_mRNA
else
    return Mock_di_mRNA
end if
```

Algorithm 2b Mock fragment Dataset

```
Input: Positive miRNA–mRNA interactions dataset
Input: Type of mRNA shuffling k_parameter (k=1 or k=2)
Output: Mock fragment negative interactions dataset
for each record ∈ positive data do
    repeat
        shuffled_extended_fragment ← uShuffle(record.site extended with 100 nts on each side, k=k_parameter)
        new_fragment ← trimming 50 nts on each side from shuffled_extended_fragment
        miRNA ← record.miRNAseq
        duplex ← RNAduplex (new_fragment, miRNA)
        if duplex.valid then
            Add (shuffled_extended_fragment, miRNA) interaction to Mock_mono_fragment dataset if k = 1, or
                                                                  Mock_di_fragment dataset if k = 2
        end if
    until valid duplex is obtained or attempt is limit reached
end for

if k=1 then
    return Mock_mono_fragment
else
    return Mock_di_fragment
end if
```

Algorithm 2c Mock fragment and miRNA Dataset

```
Input: Positive miRNA–mRNA interactions dataset
Input: Type of mRNA shuffling k_parameter (k=1 or k=2)
Output: Mock fragment and miRNA negative interactions dataset
for each record ∈ positive dataset do
    repeat
        shuffled_extended_fragment ← uShuffle (extending record.site with 100 nts on each side, k=k_parameter)
        new_fragment ← trimming 50 nts on each side from shuffled_extended_fragment
        repeat
            shuffled_miRNA←uShuffle (record.miRNAseq, k=1)
        until the sequence at positions 2–7 and 3–8 does not match with any real human miRNA
              or the attempt limit is reached
        duplex ← RNAduplex (new_fragment, shuffled_miRNA)
        if duplex.valid then
            Add (shuffled_extended_fragment, shuffled_miRNA) interaction to Mock_mono_fragment_&_miRNA dataset if k = 1, or
                                                                            Mock_di_fragment_&_miRNA dataset if k = 2
        end if
    until valid duplex is obtained or attempt is limit reached
end for

if k=1 then
    return Mock_mono_fragment_&_miRNA
else
    return Mock_di_fragment_&_miRNA
end if
```

3.3 NPS CLASH

Algorithm 3 NPS CLASH Dataset

```
Input: Positive miRNA–mRNA interactions dataset
Input: Selection method of candidate (c=MFE or c=Random)
Input: List of positive interactions full-positive-dataset (FPD)
Output: NPS CLASH negative interactions dataset
for each record ∈ positive data do
    miRNA ← record.miRNAseq
    List_of_Candidates ← []
    masked_mRNA ← MaskPositiveSites(record.mRNAseq, FPD)
    for each window of size of 75 nts on the masked_mRNA and steps of 40 nts do
        duplex ← RNAduplex (window, miRNA)
        if duplex.valid then
            Add (window, miRNA) to List_of_Candidates
        end if
    end for
    Selected_candidate_interaction ← Choose the candidate based on the selection method parameter
    Add Selected_candidate_interaction to NPS_CLASH_MFE or NPS_CLASH_Random dataset
end for

if c=MFE then
    return NPS_CLASH_MFE
else
    return NPS_CLASH_Random
end if
```

3.4 TarBase

Algorithm 4 TarBase miRNA–mRNA interactions Dataset

```
Input: TarBaseFull negative interactions dataset
Input: List of positive interactions FPD
Output: TarBase negative interaction datasets
for each record ∈ TarBaseFull do
    if record ∉ FPD then
        Add interaction to TarBase
    end if
end for

for each record ∈ TarBase do
    if record.tissue is liver then
        Add interaction to TarBase_Liver
    end if
end for

for each record ∈ TarBase do
    if record.experiment is microarray then
        Add interaction to TarBase_microarray
    end if
end for

return TarBase, TarBase_Liver, TarBase_microarray
```

3.5 CLIP non CLASH

Algorithm 5 CLIP non CLASH Dataset

```
Input: List of mRNA fragments extracted from CLIP experiment and their full mRNA mRNA-clip
Input: List of miRNAs extracted from CLIP experiment miRNA-clip
Input: List of positive interactions FPD
Output: CLIP non CLASH negative interactions dataset
all_possible_interactions ← generate all possible miRNA-mRNA pairs from mRNA-clip and miRNA-clip lists
for each pair ∈ all_possible_interactions do
    if an interaction of the same mRNA and a miRNA from the same seed family exists in FPD then
        Remove pair from all_possible_interactions
    else
        duplex ← RNAduplex(pair.mRNA, pair.miRNA)
        if duplex.valid then
            length_tail ← calculate the unpaired 5' tail of the miRNA in the duplex
            if length_tail < 5 then
                Add to CLIP_non_CLASH
            end if
        end if
    end if
end for

return CLIP_non_CLASH
```

3.6 NPS CLIP

Algorithm 6 NPS CLIP Dataset

```
Input: List of mRNA fragments extracted from CLIP experiment and their full mRNA mRNA-clip
Input: List of miRNAs extracted from CLIP experiment miRNA-clip
Input: Selection method of candidate (c=MFE or c=Random)
Input: List of positive interactions FPD
Output: NPS CLIP negative interactions datasets
for each mRNA ∈ mRNA-clip do
    List_of_Candidates ← []
    masked_mRNA ← MaskPositiveSites(mRNA.mRNAseq, FPD )
    for each window of size of 75 nts on the masked_mRNA and steps of 40nt do
        repeat
            miRNA ← choose random miRNA from miRNA-clip
            duplex ← RNAduplex (window, miRNA)
            if duplex.valid then Add (window, miRNA) to List_of_Candidates
            end if
        until valid duplex is obtained or attempt is limit reached
    end for
    Selected_candidate_interaction ← Choose the candidate based on the selection method parameter
    Add Selected_candidate_interactions to NPS_CLIP_MFE or NPS_CLIP_Random dataset
end for

if c=MFE then
    return NPS_CLIP_MFE
else
    return NPS_CLIP_Random
end if
```

References

[1] Ronny Lorenz, Stephan H Bernhart, Christian Höner Zu Siederdissen, Hakim Tafer, Christoph Flamm, Peter F Stadler, and Ivo L Hofacker. Viennarna package 2.0. *Algorithms for molecular biology*, 6(1):26, 2011.

[2] Mark Menor, Travers Ching, Xun Zhu, David Garmire, and Lana X Garmire. mirmark: a site-level and utr-level classifier for mirna target prediction. *Genome biology*, 15(10):500, 2014.

[3] Gilad Ben Or and Isana Veksler-Lublinsky. Comprehensive machine-learning-based analysis of microrna–target interactions reveals variable transferability of interaction rules across species. *BMC bioinformatics*, 22(1):1–27, 2021.

[4] Ming Wen, Peisheng Cong, Zhimin Zhang, Hongmei Lu, and Tonghua Li. Deepmirtar: a deep-learning approach for predicting human mirna targets. *Bioinformatics*, 34(22):3781–3787, 2018.
