# Supplementary material for: Benchmarking the negatives: Effect of negative data generation on the classification of miRNA-mRNA interactions
Source: PLoS Comput Biol. 2024 Aug 26;20(8):e1012385. doi: 10.1371/journal.pcbi.1012385 (PMC11379385; doi:10.1371/journal.pcbi.1012385)
Supplement: Attachment — Submitted filename: ResponseLetter_final.pdf [file pcbi.1012385.s003.pdf]

To *Plos Computational Biology*,

It is our pleasure to submit the revised manuscript "Benchmarking the negatives: effect of negative data generation on the classification of miRNA-mRNA interactions" for consideration at PLOS Computational Biology. We have carefully addressed the reviewers' comments in the manuscript, and we include a point-by-point response to the reviewers' report in the pages below (blue text).

In the manuscript file, we marked up the changes in red text.

Isana Veksler-Lublinsky

Reviewer #1

In the manuscript "Benchmarking the negatives: effect of negative data generation on the classification of miRNA-mRNA interactions" Cohen et al address the problem of negative entries, and assess approaches for comparing and contrasting one class classification vs multiple class classification.

The authors focus on animal microRNAs - details presented in the introduction and approaches point in this direction, however, this is not acknowledged by the authors.

Response: We appreciate the reviewer's valuable input. We have incorporated this crucial detail into the abstract and rephrased relevant sections of the Introduction to acknowledge its significance.

Moreover the description of the technical, yet biological, characteristics of the miRNA/mRNA interaction are not thoroughly understood and presented;

Response: We have included the determinants characterizing miRNA target interactions in various sections of the Introduction, especially within the context of surveying computational tools for predicting miRNA-target interactions.

Even though machine learning approaches are highly popular at the moment, I think it is important to thoroughly and correctly present their characteristics, and avoid high level descriptions that are not sufficiently informative to either novices or experts in the field.

Response: We thank the reviewer for this suggestion. We rewrote the paragraph about XGBoost in Methods under subsection *2.6.1. Binary classification with XGBoost*, to include its characteristics.

The description of the methods is wordy and I find it difficult to extract its essence.

Response: During our literature survey, we encountered a common issue: insufficient details regarding the implementation of methods to generate negative interactions. Therefore, our objective was to provide comprehensive technical specifics, intending this work to be a valuable resource for those seeking to replicate our methods tailored to their specific needs. Nevertheless, during revision, we condensed the text where possible to enhance clarity.

The code is made available on a github repo - this is an excellent initiative; to enhance the visibility and usefulness of the code, I suggest adding mini descriptions and comments to the code.

Response: Following the reviewer's suggestion, we improved the github repo to include a full explanation of the folders/files and the purpose of each. In addition, we added the run file for the full experiment.

For the results section, it is important to show standard ML outputs, but simple values such as accuracies are not sufficient to assess a model or interpret its efficiency.

Response: We agree with the reviewer that showing ML output is not sufficient. For that reason, we performed a variety of experiments aimed at delving deeper into the disparities between methods, beyond simple performance metrics like accuracies.
These experiments encompassed cross-dataset analysis aiming to assess the models' sensitivity to different types of negative datasets [subsection *3.2.2. Cross-datasets classification performance*].

In addition, to further understand the variability within cross-dataset results, we conducted "Two negative-classes classification" experiment [*3.2.3. Two negative-classes classification*]. Across all experiments, we utilized the SHapley Additive exPlanations (SHAP) framework to extract top important features, quantifying their influence on the model's classification.

Moreover, we characterized datasets based on base-pairing patterns and miRNA distributions. We conducted pairwise comparisons of datasets using KL distance based on their miRNA distribution. Collectively, these analyses contributed to a more profound understanding of the results and provided clearer insights into methodological differences.

The problem tackled is interesting and relevant. Unfortunately to approach doesn't capture all the information from the proposed study.

Response: We hope that the revised manuscript resolves this concern.

Reviewer #2: General comments

Positive: The interpretation of data was interesting as the authors have made an effort to investigate what the model is learning and not just taking into account accuracy metrics.

Response: We thank the reviewer for highlighting the positive aspects of our study.

Negative

The manuscript is lacking focus at large, following are some potential directions that could make the research presented actionable for the field.

a. The paper would greatly benefit from incorporating an analysis of biases against multiple negative sets.

Response: We performed this experiment previously. To make it more visible we added a new section *"Combination of negative datasets"* in the Discussion, where we explain the experiment involving the curation of a negative dataset by combining all proposed negative datasets in a balanced manner. Subsequently, we trained and tested a new model using this combined data alongside the positive set. The results revealed that the model failed to accurately classify negative interactions, and our conclusions are provided in the text.

Additionally, we carried out an experiment to assess the classifier's ability to distinguish between negative interactions derived from different methods (*3.2.3. Two negative-classes classification*). In this scenario, we anticipated challenges in distinguishing interactions with similar features compared to interactions that differ. This exploration provided valuable insights into method disparities/ similarities and even identified important features for interesting cases (Figure S15).

b. Guidelines for selecting good negatives based on the findings in the paper. Potentially, some guidelines on how to test if a model generalizes well?

Response: We thank the reviewer for this suggestion. We have added a new subsection in the Discussion section titled *"Recommendation for negative data selection and examination of new ML models".*

c. Authors used only one type of ML model (XGBoost). It would be interesting to see if the selection of ML (or even DL) model has an impact on the results.

Response: We now explicitly highlight the advantages of XGBoost and the reasons for selecting it in this study in multiple sections of the manuscript (e.g., Introduction, Methods: *2.6.1. Binary classification with XGBoost*, Discussion: *Maintained factors in dataset evaluation*).

In this study, we employed the same binary classifier trained on a fixed human positive dataset of MTIs and negative datasets generated by different methods. This approach ensures a fair and comprehensive comparison between all the negative datasets. Our objective was to thoroughly evaluate these negative datasets, gaining a profound understanding of the performance outcomes. The evaluation process included an analysis of distinctive features that differentiate between the datasets. Consequently, the machine learning model served as a tool to explore our hypothesis, and we

selected the most suitable model for our requirements based on reported literature findings. For future work, it would be intriguing to assess the impact of different models on the results, as now added in the Discussion section under “*Maintained factors in dataset evaluation*”.

d. A method used in the literature for generating negatives where positive miRNA-mRNA pairs are randomly shuffled, maybe with some constrains so that negative pairs are also not in the positive set, should be tested as it could potentially have less bias than other methods presented.

Response: We thank the reviewer for this suggestion. We actually implemented this idea in CLIP_non_CLASH method (Method 5). In this method, new interactions are generated between random miRNAs and mRNAs that were recovered in CLIP data (which indicates they are co-expressed and independently participated in some positive interactions). As also noted by the reviewer, we excluded interactions that were found in the CLASH data (positive direct miRNA-mRNA interactions) from this negative dataset.

e. The data deposited in Zenodo (https://zenodo.org/records/8199848) are not split into train and test sets making reprodcubility of the method difficult. It would be nice to have it there so results could be reproduced or new models could be trained on exactly the same data.

Response: Done (see “Availability of data and materials” section).

Review comments

Line 154 – 155: Only canonical and non-canonical interactions are considered valid in our study, both in positive and negative datasets. Why have non-seed interactions not been considered in this study?

Response**:** We revised the relevant lines in subsection “2.2. Definitions” to address this point. Briefly, we acknowledged the existing focus of works in this field on canonical and non-canonical interactions, prompting us to specifically use these types of interactions from the positive datasets. We recognize the significance of this point, given the prevalence of seed-less interactions in the data, and we agree that future studies should actively study them in various contexts, now addressed in the Discussion (“Maintained factors in dataset evaluation”).

Concerning negative interactions, we are dealing with a wide search space, and our aim is to prevent potential biases in the data, by generating high-quality negative interactions that closely resemble positive interactions. Essentially, by crafting negative interactions that include both canonical and non-canonical instances similar to positive interactions, the model can focus

on learning features related to interactions, rather than being fixated solely on the seed type.

Line 200 – 201: We chose h3 as the positive interactions dataset for our experiments. What is the reasoning to pick h3 as the positive interactions dataset?

Response: The h4 dataset poses a challenge due to its size (77,326 interactions), making it impractical to generate negative interaction datasets in certain methods (such as Mock_mRNA, Mock_miRNA, NPS_CLASH). Conversely, the h2 dataset is unsuitable as it originates from a mix of experiments where the chimers are formed by spontaneous ligation rather than a dedicated CLASH experiment.

Given the options of h3 and h1, we chose h3 arbitrarily to serve as positive data and a reference that is used for generating negative interactions. It is crucial to note that we systematically incorporated all available CLASH datasets as part of our methodology. This inclusion served to filter candidates for negative interactions, preventing instances where an interaction might be dual-classified as both positive and negative. As we acknowledged in the discussion (subsection *“Maintained factors in dataset evaluation”*), using alternative datasets and comparing the results could be a venue for future research

Line 205: We collected from the literature methods for generating negative interactions. The method by which the comprehensive literature search was performed should be explicitly stated in the materials and methods section.

Response: We thank the reviewer for this valuable suggestion. We added a supplementary figure S1 that explains the literature search methodology (referred from Methods: 2.4. Generating interactions: Negative interactions).

Line 211: (similar to, e.g., [16, 19]. missing closing parenthesis

Response: Thank you. The line is fixed now.

Line 212: a random shuffling (uShuffle with k=1 [44]). why shuffle only individual nucleotides here, when in method 2 instead both individual and pairs of nucleotides are shuffled?

Response: Initially, we implemented the Mock mRNA method using mono shuffling, resulting in relatively high performance (ACC of 0.93). As our features include both 1-mer and 2-mer mRNA composition, we realized that such high ACC occurred due to a bias in the model that learned to distinguish

between positive and negative interactions. Upon closer examination, we found that the majority of the top ten essential features are linked to the 2-mer mRNA composition. This was the reason for performing 2-mer shuffling of the mRNA. As there are no such features for miRNA, in our features set, we used only 1-mer shuffling method (see clarification in Methods: *Method 2: Mock mRNA*). We refer to this as part of the guidelines as requested above (*Discussion: Recommendation for negative data selection and examination of new ML models*).

Line 216: (the number of attempts was limited) what happens when a valid negative cannot be found after the given number of attempts

Response: In this case, when combining positive and negative data for ML experiments, imbalance is encountered and we now refer to it more clearly in *Methods: Data leakage, imbalance and splitting into training and testing sets*.

Line 310 – 311: We split the positive and negative datasets into 80% training and 20% testing according to a random state (Figure 2). Random splitting might not be sufficient for genomic sequences as it might produce data leakage - if we have two pairs of miRNA-mRNA interactions with very similar sequences and one pair goes to positive and one to negative set.

Response: We thank the reviewer for the interesting and important point.

To address the concern of data leakage, we implemented a two-step process to eliminate duplicate interactions from both positive and negative datasets. Firstly, we filtered duplicate interactions that involved the same miRNA and mRNA and formed identical duplexes. This information has been now included in (*2.4. Generating interactions, Positive interactions*)

Secondly, our ML approach is based on interaction features rather than the interacting sequences themselves. Similar sequences might form different interactions with variations in the duplex structures and their characteristics, and vice versa; different sequences can form duplexes with the same characteristics. Therefore, for each interaction in the negative datasets, we ensured that the positive dataset h3, contains no interactions with the same feature values. This additional clarification has been included in Methods (*2.7. Data leakage, imbalance and splitting into training and testing sets*).

Moreover, we acknowledge the significance of exploring sequence similarity, which remains an open aspect for future research. The challenge associated with determining a suitable threshold for sequence similarity necessitates careful consideration and in-depth understanding, making it an interesting avenue for further investigation.

Line 311 – 312: To address the class-imbalance problem, we employed the commonly used method of undersampling. what method was used for undersampling - is it just random selection, or something more elaborate?

Response: This is a very important note. We used random selection without replacement. We rewrote this line in (*2.7. Data leakage, imbalance and splitting into training and testing sets*): "To address the class-imbalance problem, we employed the commonly used method of undersampling, which involves **randomly** selecting a subset of samples from the larger class **without replacement**."

Reviewer #3: General comments

In this paper, authors show the impact of the generation of a dataset of negative miRNA-mRNA interacting pairs on the classification of Mirna-Target Interactions (MTIs), underlying the lack of a reliable benchmark dataset of negative interactions and the difficulty of classification of MTIs using existing techniques proposed for generating negative data. The paper presents a detailed set of experiments, also showing the correlation between several mirna and/or target features used for classifying MTIs and the choice of negative dataset.

The problem is well introduced and explained, and the experimental section is clear and exhaustive. However, I have some concerns mainly related to the description of the datasets used for creating the positive and negative datasets of interacting pairs. The nomenclature used is sometimes misleading, with multiple sets identified by the same term.

Response: We thank the reviewer for summarizing the contribution of our paper.

Here are my detailed comments:

1) Page 5, line 174. It's not clear to me how the complementary CLIP data differ from dataset h3. Is h3 dataset a dataset of CLASH experiment or CLIP experiments?

Response: We now provide an explanation in Methods, subsection *2.3 Data retrieval and processing: CLIP,* an explanation regarding the connection between CLIP and CLASH datasets.

2) Page 7, Figure 1.
a. Maybe it's better to add a similar picture to explain how the 3 datasets (CLASH, TarBase, CLIP) are processed.

Response: We thank the reviewer for the suggestion. We made an effort to provide comprehensive details on the processing of the three datasets in Methods (subsection 2.3 “Data retrieval and processing”), recognizing the complexity that might be challenging to convey solely through a figure.

b. I also suggest naming the positive dataset and the extended positive dataset differently instead of grouping them together (e.g. CLASH is both the name of the whole dataset including h1, h2, h3 and h4 and the name of the positive dataset h3).

Response: We thank the reviewer for this important note. We made changes in the text and Figure 1, using the term *full-positive-dataset* to denote the combined h1-h4 dataset.

c. What does "CLIP U CLASH**" mean? Is it a union of the two sets? And if so, does TarBase/CLASH** correspond to a set difference?

Response: We now changed the figure such that there is no need for using these symbols.

3) Page 7, line 211. “similar to e.g. [16,19]” there is a missing round parenthesis.

Response: Thank you. The line is fixed now.

4) Page 7, line 213. “at most one match with any known human miRNA”. This looks in contrast with what is reported in lines 210-211 where no matches with seeds of other known mirnas are allowed.

Response: We are sorry for the confusion, we rewrote this part in “*Method 1: Mock miRNA*”.

5) Page 7, lines 232-234. One of the 6 datasets is not reported in the final list: "Mock_di_mrna"

Response: We thank the reviewer for noticing this. The line in section (Method 2: Mock mRNA) is fixed now.

6) Page 8, lines 269-270. “Interactions that form… were discarded”. Please motivate this choice.

Response: We have added the motivation and clarification in the text (*Methods: Method 5: CLIP non CLASH*).

7) Page 8, lines 273-274. Again, it's not clear whether CLASH refers to all 4 datasets forming the CLASH dataset or just the h3 dataset or the extended positive datasets. I think it's better to choose 3 different names to identify these datasets.

Response: Sorry for the confusion, we have revised this part to be more clear (*Methods: Method 6: Non-positive sites CLIP (NPS CLIP)*).

8) Page 9, Section 2.5. The description of features is too short. Please specify better what is a feature and what each category represents.

In addition:
a. In Table 4, page 10, "Site accessibility" features are not well explained both in the manuscript and in the supplementary table of features. Can you provide more details about them?

Response: We have added a more comprehensive description of the features in the Supplementary material.

b. Have you considered the possibility to include in your study other features related to the stability of mirna-target duplex, e.g., the melting temperature? Or if you plan to include them in future works.

Response: In the course of our research, we sought alignment with established characteristics outlined in the literature, adhering to recognized standards. We appreciate the reviewer’s suggestions, and we plan to incorporate these insights into future studies as part of Efrat’s ongoing Ph.D., which delves deeper into this topic. Exploring additional features will indeed be intriguing. We have included this point in the Discussion section under "*Maintained factors in dataset evaluation*".

9) Page 11, lines 321-322. “Thus, we enumerated all the possible pairs of training and testing datasets: (train_i, test_j)”. This sentence is not so clear. I think it means that if we have N negative datasets the pairs are (train dataset 1, test dataset 1), (train dataset 1, test dataset 2),...,(train dataset 1, test dataset N), (train dataset 2, test dataset 1), ..., (train dataset 2, test dataset N) and so on, where training and test sets come from negative dataset.

Response: We now clarified the sentence in Methods section under *2.8. Evaluation*.

10) Page 14, Fig. 3. I think it's better to invert the colors in the color scale representing the values of metrics. Usually in a heatmap darker colors are associated to higher values.

Response: We have several heatmaps in our results and we analyzed some of them simultaneously, necessitating the use of opposite scales. Consequently, we employed darker colors to represent higher values for KL in Figure S7, while opting for the opposite scale for the other metrics to effectively illustrate the contrasts in Figure 5. Since Figure 3 represents intra-results and should match the scale of Figure 5, we maintained the same trend. same trend.

11) Page 16, lines 494-495. “while the CLIP and CLASH datasets originate from the same experiment”. I guess here with term CLASH we are talking about the h3 dataset only, which is part of the CLASH dataset. This is related to my comment above.

Response: This sentence is a general explanation of the difference between CLASH and CLIP. We refer to dataset h3 as an example used in our study. We have revised this part for clarity (Results: *3.2.1 Dataset characteristics*).

12) Page 17, Fig. 5. Also for the heatmap matrix of Fig. 5 it would be better to invert the colors in the color scale.

Response: Same answer as 10.

13) Page 20, line 628. “NPS_CLASH_20th_Percentline”. I guess it's "Percentile" and not "Percentline"

Response: Thank you. This is fixed now.
