# Supplementary material for: Benchmarking the negatives: Effect of negative data generation on the classification of miRNA-mRNA interactions
Source: PLoS Comput Biol. 2024 Aug 26;20(8):e1012385. doi: 10.1371/journal.pcbi.1012385 (PMC11379385; doi:10.1371/journal.pcbi.1012385)
Supplement: Attachment — Submitted filename: Response_letter_2.pdf [file pcbi.1012385.s004.pdf]

To *Plos Computational Biology*,

It is our pleasure to submit the revised manuscript "Benchmarking the negatives: effect of negative data generation on the classification of miRNA-mRNA interactions" for consideration at PLOS Computational Biology. We have carefully addressed the reviewer's comments in the manuscript, and we include a point-by-point response to the reviewer's report in the pages below (blue text).

In the manuscript file, we marked up the changes in red text.

Isana Veksler-Lublinsky

Reviewer #1

We are grateful to the reviewer for the insightful comments, which we believe have significantly enhanced the manuscript.

"MicroRNAs (miRNAs) are small non-codingRNAs that regulate gene expression post-transcriptionally via base-pairing with complementary sequences on messenger RNAs (mRNAs)." The statement is ambiguous. The rules of complementarity and the targeted region of the mRNA differ between plants and animals.
Based on the abstract and introduction it is still not clear whether your approach is presented on either plants or animals. Based on the characteristics of the interactions presented in the results, you are focusing on animal models.

Response: We thank the reviewer for highlighting this ambiguity. We revised the sentence for clarity. "MicroRNAs (miRNAs) are small non-coding RNAs that regulate gene expression post-transcriptionally. In animals, this regulation is achieved via base-pairing with partially complementary sequences on mainly 3' UTR region of messenger RNAs (mRNAs)".

It is important to note that in the second paragraph of the abstract, we explicitly specify our focus on human MTIs. In addition, we carefully reviewed the abstract, summary, and Introduction, making the necessary adjustments to clarify that our study deals with animal miRNAs.

"To train an ML algorithm, there is a need to supply entries from all class labels (i.e., positive and negative)."
Please note that multi-class classification is possible on a range of classic and modern ML approaches.

Response: We revised the sentence to be "To train an ML algorithm, it is beneficial to provide entries from all class labels (i.e., positive and negative)."

Moreover, the optimisation of a model can rely on partial annotations i.e. the statement "Currently, no high-throughput assays exist for capturing negative examples, hindering effective classifier construction." is misleading. You yourselves acknowledge this just few sentences below.

Response: We refined the statement and added clarification, both in the abstract and the Introduction. "Currently, no high-throughput assays exist for capturing negative examples. Therefore, current ML approaches must rely on either artificially generated or inferred negative examples deduced from experimentally identified positive miRNA-target datasets."

"This binding mode allows one miRNA to potentially regulate several hundreds of target mRNAs and for a single mRNA to be targeted by many different miRNAs."

While it is a challenge to predict the number of miRNA targets, it is agreed within the community that miRNAs do not regulate "several hundreds" targets.

Response: We refined the text. "This binding mode allows one miRNA to potentially regulate multiple target mRNAs and for a single mRNA to be targeted by multiple different miRNAs."

The description of the methods is, in places, sloppy "First, we removed the adapter sequences (as in [13]) and filtered out reads shorter than 18" - I suppose you mean 18nts? Also, the 18nts and 25nts cutoffs are arbitrary - the filtering should be data-driven.

Response: We updated the description. While we acknowledge the importance of data-driven threshold selection, we based our choices on existing literature and our experience with RNAseq data. Specifically, we referred to established length thresholds for miRNA reads, as reported in the literature (e.g., https://febs.onlinelibrary.wiley.com/doi/full/10.1111/j.1742-4658.2008.06624.x), and included this reference in the methods section for clarity.

"To obtain the mature miRNA name corresponding to the aligned region, we mapped the available mature miRNA sequences from miRBase against the precursors as well and selected the mature miRNA with the highest overlap." there are plenty of frameworks for predicting miRNAs and miRNA hairpins, and avoid being biased by the curated miRbase. Once miRNAs are predicted, it is straightforward to indicate which miRNAs are already annotated in miRbase, which are potentially novel.

Response: We thank the reviewer for this suggestion. Indeed there are tools, e.g., miRDeep and sRNAbench, that are effective for identifying miRNA hairpins from total RNA sequencing data, as they rely on the presence of both mature and star/loop sequences to define hairpins. In our case, we are working with CLIP data obtained after AGO immunoprecipitation, which contains only the functional mature sequences. Future studies could examine the possibility of identifying novel miRNAs from CLIP datasets.

The description of the 6 approaches for creating the negative dataset is not clearer. Try summarising the approach as a mini diagram, or in pseudocode.

Response: This is a very good suggestion. We summarized the methods with pseudocode, which we now provide in the Supplementary material.

The sections 2.9 to 2.12 are unclear; please link those to their usage, and, overall, support the story.

Response: We appreciate the reviewer for highlighting this confusion. To address it, we have added a title that unifies these sections along with a brief introduction to their overall usage.

"This implies that 1-mer shuffling does not maintain the original ratio distribution of 2-mer sequence composition in mRNA sequences"
I fail to see how 1-mer shuffling works.

Response: 1-mer shuffling refers to randomly rearranging individual nucleotides within a sequence while maintaining their overall frequency. This process disrupts the original sequence while preserving the frequency of each nucleotide. However, shuffling the 1-mers does not maintain the original distribution of 2-mer sequence composition. Therefore, while the frequency of individual nucleotides remains unchanged, the distribution of dinucleotides in the shuffled sequence may differ from that of the original sequence. We refined the text to enhance clarity on this point.

The panels you presented require additional support for the interpretation of outputs. All results are presented from a purely computational perspective, with no links to the biological information or its interpretation.
From a computing angle, this analysis is yet another example of applying XGBoost on a dataset - the power of your results comes from the link with the features and their subsequent interpretation.

Response: We now link between panels with computational results to their interpretation. Please refer to Figures 3 and 5 for specific examples. Additionally, the remaining panels are seamlessly integrated into the flow of the Results section.

Reviewer #2: The authors replies to my comments have been satisfactory. The changes made have substantially improved the manuscript.

Reviewer #3: Authors have addressed my comments.
