# Supplementary material for: Benchmarking the negatives: Effect of negative data generation on the classification of miRNA-mRNA interactions
Source: PLoS Comput Biol. 2024 Aug 26;20(8):e1012385. doi: 10.1371/journal.pcbi.1012385 (PMC11379385; doi:10.1371/journal.pcbi.1012385)
Supplement: Attachment — Submitted filename: Response_letter_3.pdf [file pcbi.1012385.s005.pdf]

To *Plos Computational Biology*,

It is our pleasure to submit the revised manuscript “Benchmarking the negatives: effect of negative data generation on the classification of miRNA-mRNA interactions" for consideration at PLOS Computational Biology. We have carefully addressed the reviewer’s comments in the manuscript, and we include a point-by-point response to the reviewer’s report in the pages below (blue text).

In the manuscript file, we marked up the changes in red text.

Isana Veksler-Lublinsky

Reviewer #1

This paper includes a comprehensive study in order to evaluate and compare various approaches for computationally generating negative examples for miRNA-target interactions that can be used for training relevant machine learning (ML) models. Since there are no high-throughput assays for capturing negative interactions, this research should be very helpful. The results suggest among others that it is important to computationally generate such interactions for ML training and provide detailed guidelines related to how to generate such negative examples computationally. This is a helpful study.

Response: We thank the reviewer for summarizing the contribution of our paper.

1) I understand that all the analyzed data are from experiments in humans. This may lead to bias in the conclusions. It will be great to analyze a dataset from a different organism (e.g. mouse) or at least discuss this drawback of the experiment in the discussion section.

Response: This is a very good point. In this study, to achieve our goals and perform a rigorous comparison between methods, we needed to fix certain parameters, including the organism of study.

Our previous experiments with MTI across different species (https://doi.org/10.1016/j.heliyon.2024.e28000 and https://doi.org/10.1186/s12859-021-04164-x) have demonstrated that MTI rules are transferable between evolutionary close species. Therefore, we speculate that our findings in humans may also extend to at least closely related species, such as mice.

To clarify that for readers, we have revised the relevant section in the discussion, “Maintained factors in dataset evaluation,” to emphasize that our conclusions are made based on human data and that further research is needed to determine whether these findings extend to other species.

2) I suggest adding to the discussion section a paragraph regarding the generalization of the reported study to other type of data. For example, instead of miRNA-mRNA interactions, protein-DNA interactions: what should we do in such a case.

Response: Thank you for your insightful suggestion. We agree that discussing the generalization of our study to other types of data, such as protein-DNA interactions, would enhance the manuscript.

We have now added a new paragraph to the discussion: “Extension to other biological interaction data”

3) " Combination of negative datasets
Interestingly, when creating a negative dataset composed of a balanced mixture of all negative datasets proposed in this study, and training/testing a new model with this data along with the positive set, the model fails to classify the negative interactions correctly. This reiterates the insight that the negative interactions are biased by the specific strategy of each method, preventing the model from identifying a pattern that characterizes all negative interactions on the one hand and distinguishes them from the positive interactions on the other."

This is indeed a surprising result. I wonder if with a different model and strategy the negative interactions will be classified correctly? it may also suggest that the current model is "overfitted" to the computationally generated negative interactions; and that if/when experimental data of negative interactions will be generated the model will not classify them correctly. This issue should at least be discussed.

Response: Throughout the manuscript, we thoroughly analyzed multiple methods for generating negative datasets to understand the differences and their impact on performance.

We conducted several experiments to achieve this. In section "3.1 Intra-dataset analysis identifies inherent biases in some strategies for negative data generation," we demonstrated that certain methods are biased and are therefore not recommended.

In section "3.2 The cross-dataset analysis demonstrates the models' sensitivity to negative data in the MTI classification task," we showed that some, but not all, models could generalize from one dataset to another, correctly classifying both negative and positive interactions.

To gain a deeper understanding of the differences in the methods' characteristics, we conducted an experiment detailed in section "3.2.3 Two negative-classes classification." Here, we mixed pairs of negative datasets and evaluated the classifier's ability to distinguish between them. For some pairs, the classifier could easily distinguish between the datasets due to their differing characteristics.

In the discussion section, we refer to an additional experiment where we mixed the positive dataset with all negative datasets generated using the eight methods introduced in this study. The results showed that the classifier could not find a consistent pattern that differentiated all negatives from the positive data, reiterating the conclusion from our previous experiments. We did not exhaustively examine other strategies for combining different negative datasets.

The reviewer’s point that other strategies of combining different negative datasets could perform better is important and very interesting and should be studied in future research. Future studies could explore ensemble classification strategies, majority voting, etc. We now address this issue in the discussion by adding the following sentence: “It is possible that other strategies, including ensemble methods for combining models trained on different negative datasets, would yield better performance and should be examined in future studies.”.

Following this study's current approach, i.e., choosing a particular method for negative dataset generation, we provide guidelines in the "Discussion: Recommendation for Negative Data Selection and Examination of New ML Models" for evaluating the generalizability of ML models for MTI classification, including on new unseen experimental data.
